# Anoxygenic photosynthesis with emphasis on green sulfur bacteria and a perspective for hydrogen sulfide detoxification of anoxic environments

**DOI:** 10.3389/fmicb.2024.1417714

**Published:** 2024-07-11

**Authors:** Ivan Kushkevych, Vít Procházka, Monika Vítězová, Dani Dordević, Mohamed Abd El-Salam, Simon K.-M. R. Rittmann

**Affiliations:** ^1^Department of Experimental Biology, Faculty of Science, Masaryk University, Brno, Czechia; ^2^Department of Plant Origin Foodstuffs Hygiene and Technology, Faculty of Veterinary Hygiene and Ecology, University of Veterinary Sciences, Brno, Czechia; ^3^Department of Pharmacognosy, Faculty of Pharmacy, Delta University for Science and Technology, Gamasa, Egypt; ^4^School of Pharmacy and Biomolecular Sciences, Royal College of Surgeons in Ireland, Dublin, Ireland; ^5^Archaea Physiology & Biotechnology Group, Department of Functional and Evolutionary Ecology, Universität Wien, Wien, Austria

**Keywords:** bacterial photosynthesis, anoxygenic bacteria, bacterial physiology, microbiology, anaerobes, biotechnology

## Abstract

The bacterial light-dependent energy metabolism can be divided into two types: oxygenic and anoxygenic photosynthesis. Bacterial oxygenic photosynthesis is similar to plants and is characteristic for cyanobacteria. Bacterial anoxygenic photosynthesis is performed by anoxygenic phototrophs, especially green sulfur bacteria (GSB; family *Chlorobiaceae*) and purple sulfur bacteria (PSB; family *Chromatiaceae*). In anoxygenic photosynthesis, hydrogen sulfide (H_2_S) is used as the main electron donor, which differs from plants or cyanobacteria where water is the main source of electrons. This review mainly focuses on the microbiology of GSB, which may be found in water or soil ecosystems where H_2_S is abundant. GSB oxidize H_2_S to elemental sulfur. GSB possess special structures—chlorosomes—wherein photosynthetic pigments are located. Chlorosomes are vesicles that are surrounded by a lipid monolayer that serve as light-collecting antennas. The carbon source of GSB is carbon dioxide, which is assimilated through the reverse tricarboxylic acid cycle. Our review provides a thorough introduction to the comparative eco-physiology of GSB and discusses selected application possibilities of anoxygenic phototrophs in the fields of environmental management, bioremediation, and biotechnology.

## 1 Introduction

**Figure 1:**
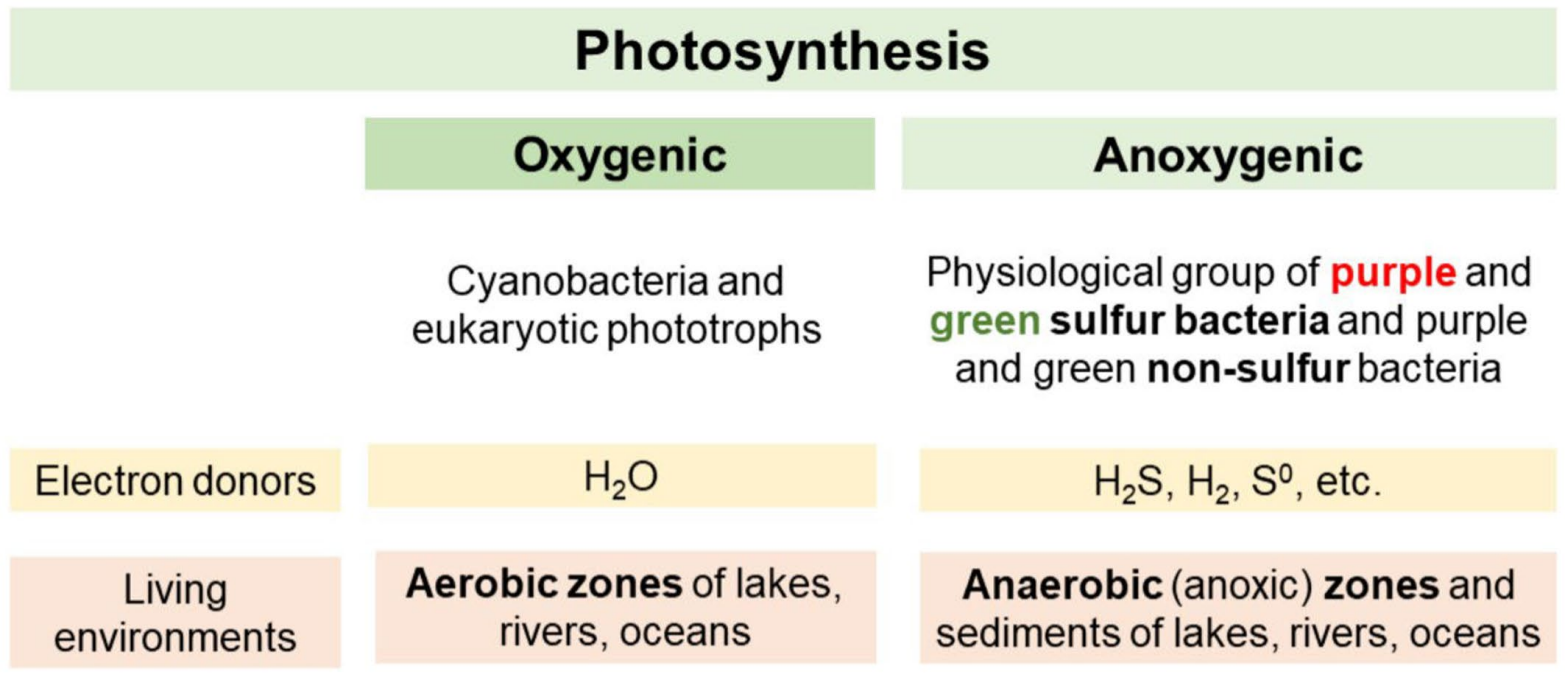
Differences between oxygenic and anoxygenic phototrophs.

In bacteria the mechanisms of photosynthesis differ from those of eukaryotes (Kolber et al., 2000). Cyanobacteria are the only bacteria that use oxygenic photosynthesis, in which water acts as an electron donor being oxidized to molecular oxygen (O_2_) (Percival and Williams, 2014; Sánchez-Baracaldo and Cardona, 2020). An advantage is that H_2_O is already abundantly available in many ecosystems, but this H_2_O-dependent photosynthetic reaction is energetically very demanding, because water is a stable compound. However, it also means a large yield of energy can be obtained. These advantages led to a significant expansion of oxygenic phototrophs in radiation events during evolution (Percival and Williams, 2014; Lazar et al., 2022). The differences between oxygenic and anoxygenic phototrophs are summarized in Figure 1.

**Figure 2:**
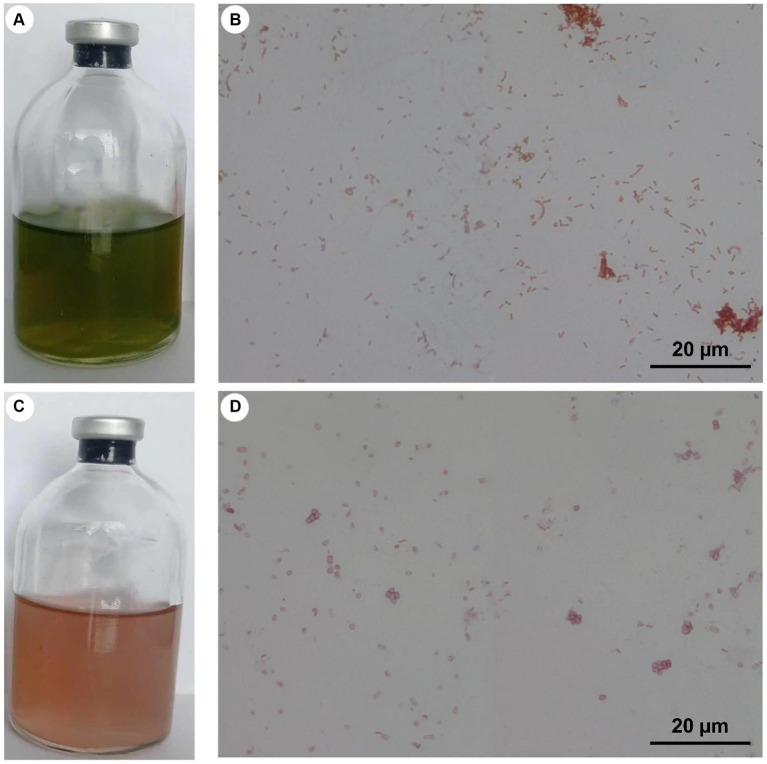
Cultures of GSB [*Cbi. limicola* **(A,B)**] and PSB [*Thiocapsa* sp. **(C,D)**], with light microscopic images at a magnification of 1,000 times **(B,D)**. These cultures were enriched and purified in the authors’ lab but have not yet been microbiologically characterized.

Both of these groups can be present in soil or freshwater and are Gram-negative bacteria. In anaerobic phototrophic bacteria, different mechanisms for energy conservation are known (Hanada, 2016). Different electron donors can be used, for example molecular hydrogen (H_2_) or reduced metal ions (Unden, 2013). A specific group of anaerobic organisms are phototrophic sulfur bacteria. These organisms use various reduced forms of sulfur, most often hydrogen sulfide (H_2_S), as an electron donor (Madigan and Jung, 2009; Swingley et al., 2009). Anoxygenic sulfur bacteria can be divided into two main groups according to their pigmentation: green sulfur bacteria (GSB) and purple sulfur bacteria (PSB) (Kushkevych et al., 2021b). Macroscopic and light microscopic images of cultures of GSB and PSB are shown in Figure 2.

GSB and PSB occur lower in the water column compared to oxygenic phototrophs (Kushkevych et al., 2021a). Overall, it can be asserted that GSB are adapted to the deeper regions of the water column or soil than PSB (van Niel, 1932; Manske et al., 2005). GSB can grow better under low light intensity, exhibit greater sensitivity to oxidizing environments compared to PSB, and, conversely, demonstrate tolerance to higher concentrations of H_2_S (Hunter et al., 2009). Another difference between the two types of sulfur bacteria concerns their way to store sulfur, which can be a product of H_2_S oxidation. H_2_S can be toxic for other group of microbial communities (Černý et al., 2018; Dordević et al., 2020; Kushkevych et al., 2020). PSB store sulfur in intracellular globules, while GSB deposit sulfur extracellularly (Woese et al., 1985; Bryant and Frigaard, 2006; Frigaard and Dahl, 2008).

The biotechnological use of anoxygenic phototrophs has only been the topic of few studies (Struk et al., 2023). However, H_2_S is a frequent contaminant of wastewater (Kushkevych et al., 2017, 2018a), whether industrial or municipal, and of natural gas or biogas (Struk et al., 2019). Thus, biotechnological removal of H_2_S through microbial oxidation could be a suitable alternative to physico-chemical cleaning methods (Struk et al., 2020). The product is most often elemental sulfur, which is insoluble in water, so it can be easily separated (Saeid and Chojnacka, 2014). Another interesting feature for putative biotechnological applications is the accumulation of glycogen during photosynthesis, e.g., known from *Chlorobium limicola*, a member of the GSB. Moreover, the use of captured light energy to generate electrical energy through microbial electrochemical cells is also being considered (Figueras et al., 2006).

This review aims to summarize knowledge about the microbiology and physiology of GSB, especially the genus *Chlorobium*. First, a general introduction of physiology, phylogeny, and taxonomy is provided. Then the process of anoxygenic photosynthesis from the capture of a light quantum through the transport of an excited electron and the replenishment of electrons from H_2_S or other electron donors is discussed. Finally, examples of practical applications of these anaerobic phototrophs will be presented, especially focusing on H_2_S removal, to evaluate the current status of their biotechnological potential.

## 2 General characteristics of green sulfur bacteria

### 2.1 Taxonomy

**Figure 3:**
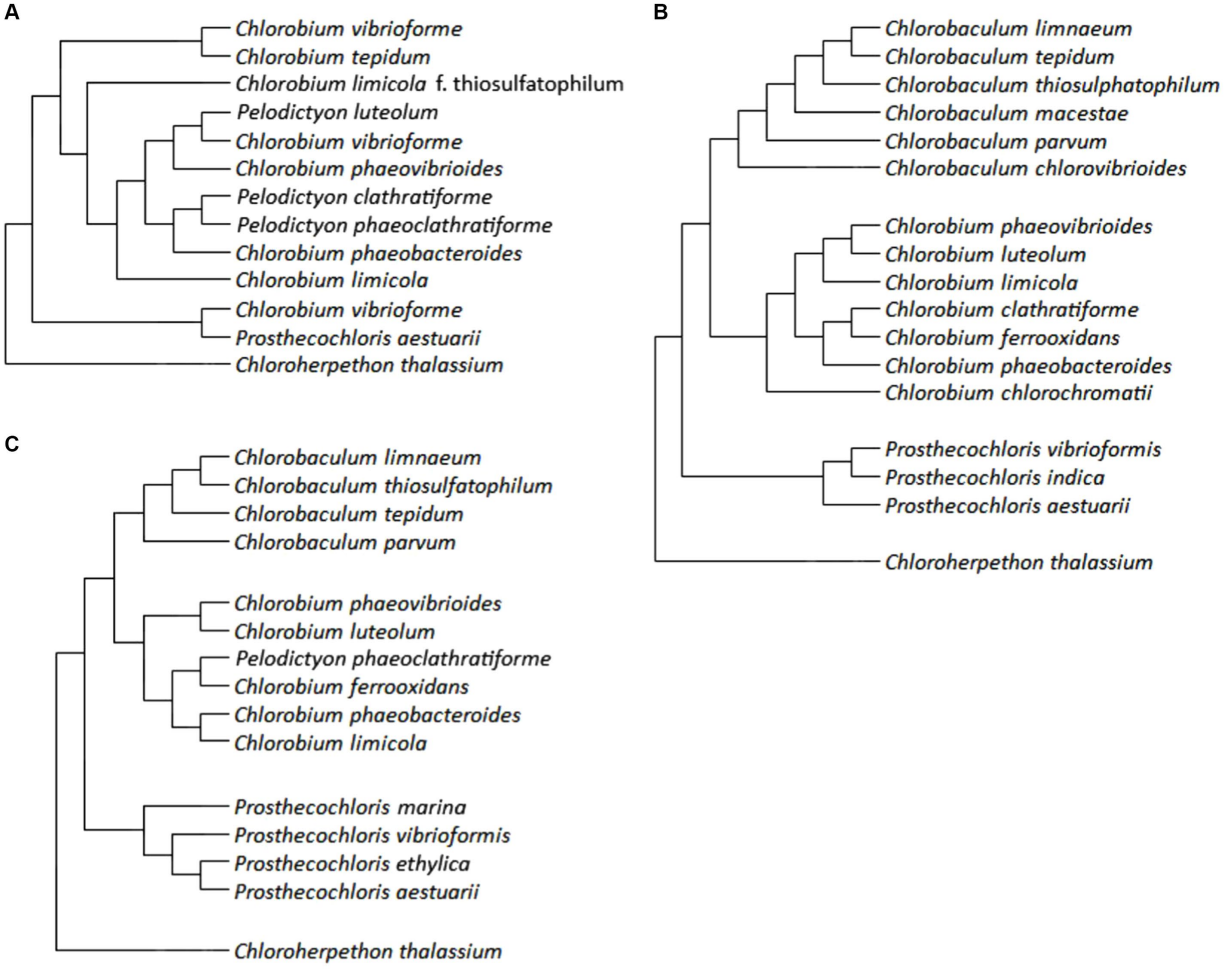
Phylogenetic trees of GSB: panel **(A)** is constructed using data by Overmann (2015), panel **(B)** is created from data by Imhoff and Thiel (2010), and panel **(C)** is based on results of Bello et al. (2022).

The initial system of GSB taxonomy was based on morphological features: color (brown or green, or the content of the carotenoids isorenieratene and chlorobactane), the formation of gas pockets and the ability to use thiosulfate as an electron donor for photosynthesis. The phylogenetic trees of GSBs (Figure 3) were constructed and discussed using various literature papers of Overmann (2015), Imhoff and Thiel (2010), and Bello et al. (2022).

A phylogenetic tree based on 16S rRNA gene sequences of GSBs was generated by Overmann and Tuschak (1997). Overmann (2015) who added *Chloroherpethon thalassium* to this tree (Figure 3A), also compared the tree to other biochemical and cytological traits such as the GC pair content, growth on saline medium, fatty acid composition, carotenoid composition, and gas vesicles presence. Their conclusion was that the GC pair content and fatty acid composition are similar for closely related species based on 16S rRNA gene sequence and therefore might give us information about the phylogeny. However, carotenoids and gas vesicles did not correlate with the results from 16S rRNA gene sequencing (Overmann, 2015).

Imhoff and Thiel (2010) revised the phylogeny of GSB based on the sequences of genes of 16S rRNA and of the FMO protein (gene *fmoA*) by taking the evolutionary significance of individual positions into account. An additional criterion was the content of GC pairs (Imhoff and Thiel, 2010). Although GSB are an ecologically defined group, they also belong to the same phylogenetic group, referred to as the *Chlorobi*. Only one chemotrophic representative, *Ignavibacterium album* has been found in this phylum.

However, within the class *Chlorobia*, which could be considered as a synonym for GSB, only phototrophic representatives are known. This class is phylogenetically distant from other bacteria (<83% similarity in the 16S rRNA gene sequence), the mutual relationship of its representatives (>88% similarity in the 16S rRNA gene sequence) corresponds to the fact that it includes a single family: *Chlorobiaceae*. Within this family, the existence of four genera was confirmed: *Chlorobaculum*, *Chlorobium*, *Chloroherpethon*, and *Prosthecochloris*. The type species were selected for these genera: *Chlorobium limicola* (at the same time it is the type species for the entire family), *Cba. tepidum*, *Ptc. aestuarii*, and *Chp. thalassium*. The only studied representative of the genus *Chloroherpethon* is a phylogenetically basal species of obligate marine bacteria (Imhoff and Thiel, 2010).

Imhoff and Thiel introduced the following changes (Imhoff and Thiel, 2010). *Chlorobium limicola* f. thiosulfatophilum was reclassified as *Cba. thiosulfatophilum* (strains with a GC pair content of around 58%) and *Cbi. limicola* (strains with a GC pair content of around 52%) (In some of the studies cited in their work, it is not possible to distinguish which of these species it is, so the invalid name corresponding to the relevant study has not been consiered). *Chlorobium vibriofirme* was divided into five species: *Cbi. luteolum*, *Cbi. phaeovibrioides*, *Ptc. vibrioformis*, and *Chlorobaculum* sp.; *Cbi. vibriofirme* f. thiosulfatophilum was reclassified as *Cba. parvum*. *Pelodictyon clathratiforme* was reclassified as *Cbi. clathratifirme*; since it was the type species of the genus *Pelodictyon*, this means the extinction of this genus and the reclassification of its other species: *Pld. luteolum* → *Cbi. luteolum*, *Pld. phaeoclathratiforme* → *Cbi. clathratiforme*. Selected strains of *Cbi. phaeobacteroides* were reclassified as *Cbi. limicola* or as *Cba. limnaeum* sp. nov. *Chlorobium tepidum* was reclassified as *Chlorobaculum tepidum*. *Chlorobium chlorovibrioides* was reclassified as *Cba. Chlorovibrioides*. Figure 3B shows the phylogenetic relationships of selected described species. Several different isolates from some species were examined, all mentioned species appeared as monophyletic in the analysis. Three representatives of the genus *Flexibacter* were used as an outgroup (Imhoff and Thiel, 2010). In Figure 3C, an updated phylogenetic tree of 282 conserved proteins is shown. This phylogenetic tree was then compared with phylogenetic trees constructed according to DNA gyrases A and B (*GyrA*, *GyrB*), DNA polymerase A (PolA), DNA helicase UvrD and 16S rRNA. Several members of the Bacteroidetes tribe were used as an outgroup. Furthermore, suitable sequences for the determination of GSB, the so-called conservative signature indels (CSIs), were selected. *Chlorobiaceae* were confirmed to be uniformly non-motile, anaerobic, photoautotrophic bacteria, using reduced forms of sulfur as electron donors for photosynthesis; in the genus *Chloroherpethon* it is bioinformatically predicted that the organisms ought to grow photoheterotrophically. Moreover, it lacks the genes of the DSR and Sox groups. Unexpectedly, the study recognizes *Pld. phaeoclathratiforme* as a valid name, even though it phylogenetically classifies this species within the genus *Chlorobium*. The phylogenetic relationships of some species are slightly different compared to the previous study, for example the relationship of *Cba. limnaeum*, *Cba. tepidum*, and *Cba. thiosulphatophilum*, or the position of *Cbi. limicola* within the tree. However, the described species and genera remain valid. Probably the biggest change introduced in their study is the allocation of the genus *Chloroherpethon* to the new family *Chloroherpethonaceae* together with the new genus *Candidatus* thermochlorobacter (Bello et al., 2022).

Based on the sequence similarity of the operational genes, the *Bacteroidetes* phylum was identified as the closest relative of the phylum *Chlorobi*. An unusually high number of proteins (about 12%) are similar to archaeal proteins. Since *Chlorobi* are considered one of the basal groups within bacteria, they may have retained genes from the common ancestor of bacteria and archaea that phylogenetically later evolved groups have lost. Another possibility is the horizontal transfer of these genes from archaea (Eisen et al., 2002).

### 2.2 Genomic studies

The thermophilic GSB *Cba. tepidum* was the first GSB with a sequenced genome harboring a genome size is 2,154,946 bp (Bryant, 2019). The organism is predicted to harbor 20 genes for regulation of transcription and 19 genes encode for aminoacyl-tRNA synthetases (Rubio Gomez and Ibba, 2020). The gene for the asparagine synthetase is missing and the gene for the glutamine synthetase is encoded but the holoenzyme is putative nonfunctional due to the absence of another necessary enzyme. *Cba. tepidum* probably uses aspartate and glutamate instead of asparagine and glutamine and converts them to the corresponding amides posttranslationally. A gene for the enzyme GatABC, which could transfer the amide group from glutamine, was found (Eisen et al., 2002), which indicated that this gene is essential for the amino acid metabolism of this organism. Although GSB are found in reducing and light-reduced environments, where DNA damage does not occur very often, *Cba. tepidum* has several DNA repair mechanisms: two UvrA endonucleases, DNA polymerase B, and a class II photolyase homolog. Genes for antioxidant enzymes are also present: superoxide dismutase, rubredoxin, oxygen oxidoreductase, and cytochrome *bd* quinol oxidase. Enzymes for the assimilation of organic substances are generally absent; only one such operon was found with genes that could enable import, phosphorylation and further processing of maltose and other maltooligosaccharides. Some components of the phosphotransferase system were also found, but incomplete; these may rather have a regulatory function. The usual source of nitrogen is NH_4_^+^, but genes for molecular nitrogen (N_2_) fixation have also been detected. If this species is able to fix N_2_ it might assist them to thrive in habitats that lack other forms of nitrogen. Furthermore, many genes for the transport of various metal ions and maintenance of their concentration, specifically six homologs of the ArsA enzyme, for ATP-dependent arsenite out-port have been identified (Eisen et al., 2002).

In a study by Davenport et al. (2010), comparative genomics of several species of all four genera of GSB were examined (Eisen et al., 2002). All genomes consisted of a single circular DNA molecule, which was 1.9–3.3 Mbp long. The GC pairs constituted a median of 50% of the content, with 87% of sequences being coding, aligning with the typical characteristics observed in bacteria. However, the share of regulatory sequences and therefore the size of the genome is smaller than in *Firmicutes* or *Pseudomonadota*. Moreover, CRISPR elements were detected. The study also dealt with pseudogenes created by displacement mutation. Due to the high content of coding sequences, not many pseudogenes were found. Furthermore, a map of tetranucleotide use and frequent 8–14 oligomers was constructed. Adhesins were among the largest genes that were identified. The arrangement of genes on the chromosome is not conserved except for closely related taxa (Davenport et al., 2010).

In another study, the genomes of populations of GSB from various locations in Europe and North America were examined. A total of 509 genomes were obtained. Based on average nucleotide identity (ANI), the genomes were arranged into 71 metagenomic operational taxonomic units and a phylogenetic tree was constructed. As expected, the presence of genes for glycolysis, gluconeogenesis, the reverse tricarboxylic acid cycle, and the synthesis of chlorophylls and bacteriochlorophylls was conserved. Nitrogenase genes were highly prevalent as well, especially NifH (nitrogenase iron protein). Several genes for H_2_S oxidation and for other photosynthetic electron donors were found. Sometimes more than one of these genes was present per genome. The vast majority of sulfur related genes that were found are sulfide: quinone reductases, reverse dissimilation reductases of sulfite and flavocytochrome *c*. Moreover, thiosulfohydrolases were present only in a small part of units from the genus *Chlorobium*. They were far more represented in the genus *Chlorobaculum*. Hydrogenases were also detected, however the ecological significance of H_2_ oxidation in GSB has generally not yet received much attention. Homologs of *cyc2*, which encodes cytochrome *c*—capable of oxidizing iron ions—were unexpectedly frequent in the genus *Chlorobium*. It is therefore possible that GSB play a significant role in the geobiochemical cycle of iron. Other putatively confirmed pathways were the pentose phosphate and ornithine cycles (Garcia and Kim, 2021).

### 2.3 Cell structure and chlorosomes

The cells of different GSB possess different morphologies: they appear as cocci (often in chains), rods, curved or spiral cells. Some species produce gas vesicles. The genus *Chloroherpethon* has a special structure: the cells are shaped like long flexible fibers and can move by gliding. Other GSB are listed as immobile (van Niel, 1932; Overmann, 2015). Some representatives of GSB, such as *Cbi. thiosulfatophilum*, are able to form polyphosphate granulae. Up to three electron-dense granules per cell were observed using electron microscopy. The formation of polyphosphate granules was monitored during growth. In the lag phase, polyphosphate was rather consumed. During the exponential and stationary phases, polyphosphate was stored (Hughes et al., 1963).

Green sulfur bacteria of the genus *Prosthecochloris* may form appendages, which are termed prosthecae. Prosthecae are protrusions of the cytoplasmic membrane that provide a larger area for photosynthetic processes. Unlike prosthecae of some heterotrophs, which help them to adapt to an oligotrophic environment, the formation of prosthecae is light-dependent: when the light flux is low, cells produce more prosthecae to capture more photons. The morphological changes of *Prosthecochloris aestuarii* depending on illumination were studied. These changes were observed using scanning and transmission electron microscopy. At a light quanta flux intensity of 0.5 μmol m^−2^ s^−1^, the average cell length was 900 nm and the average diameter was 231.8 nm; at an intensity of 100 μmol m^−2^ s^−1^, the average cell length was 1,300 nm, but the average diameter was only 98 nm (Guyoneaud et al., 2001).

Chlorosomes are an unusual type of light-harvesting antennae that are found only in GSB and green non-sulfur bacteria (*Chloroflexi*) (Chen et al., 2020). Chlorosomes are the most efficient light-harvesting complexes known, capable of working even at very low light intensity (less than 4 μEinst m^−2^ s^−1^), which enables to thrive deep in the water column or in sediments (Overmann et al., 1992a). Chlorosomes have a unique organization: instead of interactions between proteins and pigments, they are held together by interactions directly between pigment molecules, which, in addition, are assembled into supramolecular units by themselves without any protein stabilization. Chlorosomes are quite large since they contain up to hundreds of thousands of bacteriochlorophyll molecules, have an ellipsoidal shape with the longest diameter up to 1,800 Å and the shortest up to 500 Å. They are found attached beneath the cytoplasmic membrane. In *Ptc. aestuarii* the density of chlorosomes and the ratio of the membrane perimeter to its area reached the highest values at mean light intensity of 5 μmol m^−2^ s^−1^. The main pigments in *Ptc. aestuarii* were identified using HPLC as bacteriochlorophyll *c*, chlorobactane and hydroxychlorobactane. The specific content of bacteriochlorophyll *c* and carotenoids increased with decreasing light intensity as well as the development of new prosthecae in order to provide sufficient rate of photosynthesis. The ratio of the content of bacteriochlorophyll *c* to carotenoids had a value of around 12 at light intensity of 2.5 μmol m^−2^ s^−1^ and less, and increased up to fourfold at higher intensities to avoid photodamage of chlorosomes (Guyoneaud et al., 2001).

The internal organization of chlorosomes of *Cba. tepidum* was studied using X-ray scattering and electron microscopy (Pšenčík et al., 2004). This study disproved the theory that pigments are organized into rods: such rods would have to be compressed to an unimaginable density. Instead, a lamellar model of pigment organization was created. The results indicate that the lamellae wave is perpendicular to the longest axis of the chlorosome. The rod-like elements, previously observed with electron microscopes, may have been created by splitting the lamellae along these waves during the sample preparation. The lamellae consist of bacteriochlorophylls and the hydrophobic space between them is filled by carotenoids. There are various hypotheses regarding the particular relative arrangement of the pigment molecules; X-ray crystallography is usually used to evaluate the structure, but it is not suitable for chlorosomes due to their size and diversity. The chlorosomes are attached to the cytoplasmic membrane via the basal plate which contains bacteriochlorophyll *a*, bound by the CsmA protein into a paracrystalline structure. The basal plate allows light-excited electrons to leave the chlorosome and be transferred to the photosynthetic reaction center. The rest of the chlorosome is enveloped in a lipid monolayer with proteins (Pšenčík et al., 2004). The structure of chlorosomes can be influenced by the environment. The influence of the carbon source (bicarbonate alone, with acetate or with pyruvate) and temperature (30°C, 50°C, increase from 30 to 50°C, decrease from 50 to 30°C) on growth of *Cba. tepidum* cultures was monitored. In the case of the growth of bicarbonate with pyruvate and at decreasing temperatures, the slowest growth was observed, accompanied by the reduction of chlorosomes. A further study of these chlorosomes showed more frequent ethylation of positions 8 and 12 of bacteriochlorophyll *c* (which leads to blue-shift of absorption maxima) or a smaller number of reaction centers in the chlorosome (15–25 compared to the usual 25–45). Under stress conditions, cells likely lack the energy to synthesize complete chlorosomes (Tang et al., 2013). Later, surface-rendered representation and intracellular chlorosome organization of *Cba. tepidum* cells was performed using cryo-electron tomography to reveal the distribution of chlorosomes in 3D in an unperturbed cell. Furthermore, the connecting elements between chlorosomes and the cytoplasmic membrane as well as the distribution of reaction centers in the cytoplasmic membrane has been revealed (Kudryashev et al., 2014).

### 2.4 Pigments

**Figure 4:**
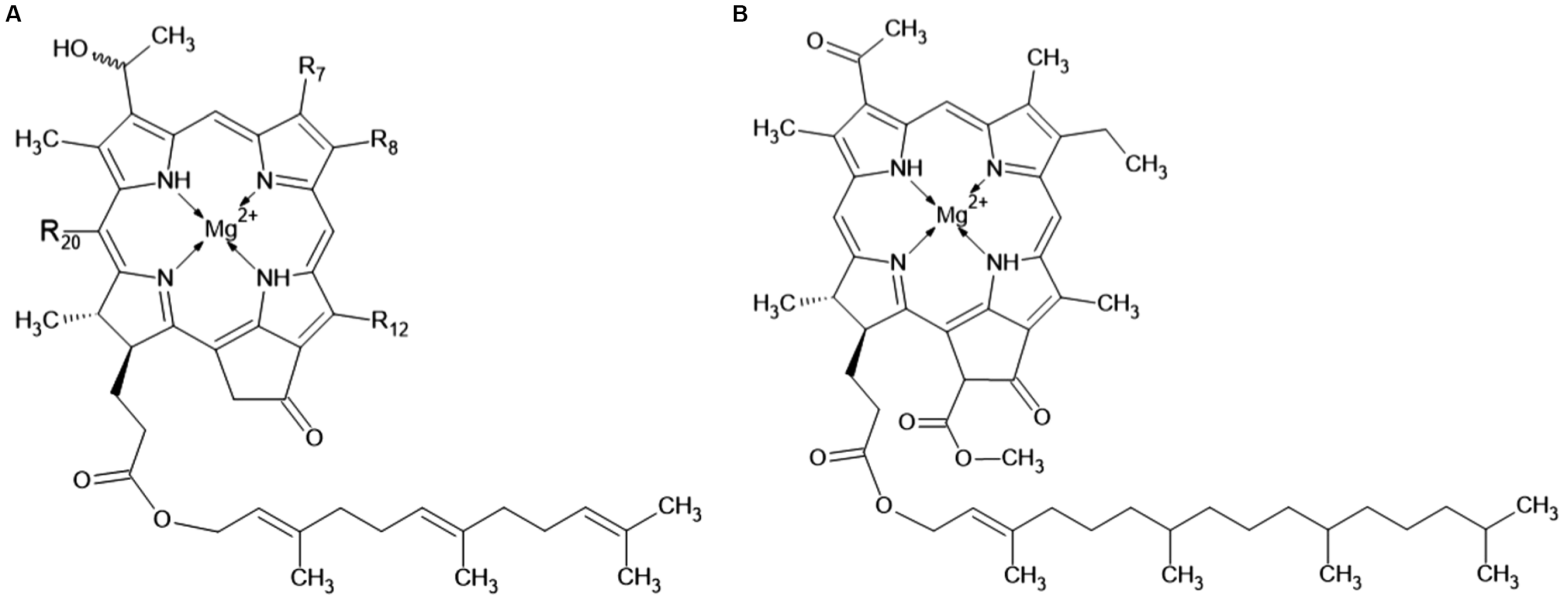
Chemical structure of the chlorobial bacteriochlorophylls: panel **(A)** indicates bacteriochlorophylls *c*, *d*, *e*, and *f*, and panel **(B)** shows bacteriochlorophyll *a*.

The main pigments of the light-collecting antennas in chlorosomes are bacteriochlorophylls *c*, *d* or *e*. These are the so-called chlorobial bacteriochlorophylls (Figure 4), known only in organisms bearing chlorosomes, i.e., in GSB and members of the *Chloroflexi* phylum. GSB usually contain only one of these pigments. Figure 4A shows the structure of these pigments. R_8_ is ethyl, propyl, isobutyl or neopentyl, R_12_ is methyl or ethyl. The differences between them are these:

- bacteriochlorophyll *c*: R_7_ = –CH_3_, R_20_ = –CH_3_
- bacteriochlorophyll *d*: R_7_ = –CH_3_, R_20_ = –H
- bacteriochlorophyll *e*: R_7_ = –CHO, R_20_ = –CH_3_
- bacteriochlorophyll *f* (not yet observed): R_7_ = –CHO, R_20_ = –H

Otte et al. (1993) studied bacteriochlorophylls in *Ptc. vibrioformis* (strain 6030, DSM 260^T^) and *Cbi. phaeovibrioides* (strain 2631). Bacteriochlorophyll *c* and bacteriochlorophyll *d* were found in *Ptc. vibrioformis*. *Cbi. phaeovibrioides* was found to possess bacteriochlorophyll *e*. Only 1–2% of the pigments are bacteriochlorophyll *a* (Figure 4B). Its primary function is not the capture of the light quantum, but the transport of the captured energy through the basal plate (see chapter 2.1) (Orf and Blankenship, 2013).

After the release of the *Cba. tepidum* genome, orthologs of all genes for the synthesis of bacteriochlorophyll *a* from protoporphyrin IX were found, which shows that this species is not auxotrophic for this compounds (Eisen et al., 2002). In the synthesis of bacteriochlorophyll *c*, only three of the enzymes that are also part of the pathway for the synthesis of bacteriochlorophyll *a* are apparently used: magnesium chelatase, vinyl reductase, and protochlorophyllide reductase, whereof the latter three variants are known. The remaining genes for the bacteriochlorophyll *c* synthetic pathway were identified as paralogs of various other genes for tetrapyrrole metabolism. Two genes for cobalt chelatases and other genes for enzymes that should be able to synthesize vitamin B_12_ were also found. In laboratory conditions, however, this vitamin is usually added to the growth medium as a source for the tetrapyrrole ring. A metaproteo-genomic study of a GSB from Antarctica showed that a complete synthetic pathway for glutamate-derived tetrapyrroles that are present in this bacterium (Ng et al., 2010).

At reduced light intensity, alkylation of bacteriochlorophyll in positions 18 and 20 is increased, which leads to a shift of the absorbance to higher wavelengths. The carbon source for this alkylation is S-adenosylmethionine. Five genes for proteins from the BchE/P-methylase family, which are possible catalysts of this alkylation, were found in the genome of *Cba. tepidum* (Eisen et al., 2002).

**Figure 5:**
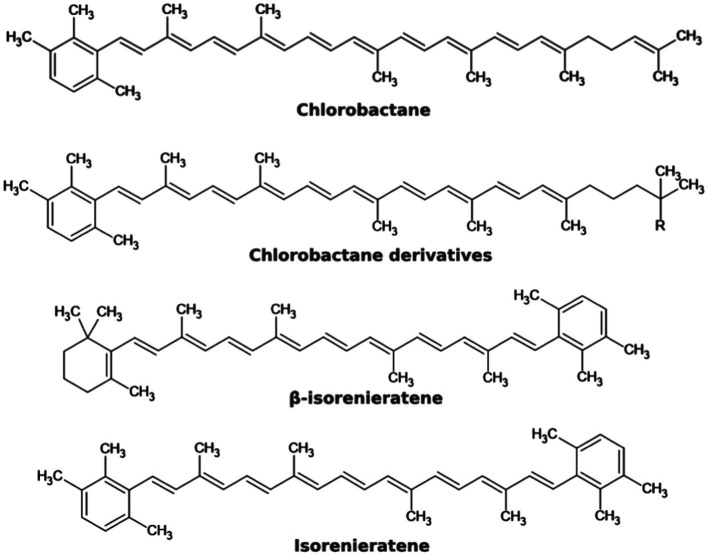
Chemical structures of some carotenoids that may occur in GSB.

In addition to tetrapyrroles, GSB also contain carotenoids. These have the highest absorbance in wavelengths 400–550 nm and transmit the captured energy toward the photosynthetic reaction center. They also protect the cell from the harmful effects of radiation and free radicals (Frank and Cogdell, 1996; Maresca et al., 2008). Furthermore, carotenoids can have a structural function. More than 90% of carotenoids in *Cba. tepidum* cells are located in chlorosomes. *Chlorobaculum limnaeum*, a brown colored representative is adapted to lower light intensity, mainly contains isorenieratene or β-isorenieratene. In *Cba. tepidum*, the most abundant carotenoid is chlorobactane, or its derivatives, the structure of which is shown in Figure 5: 1′,2′-dihydrochlorobactane, hydroxychlorobactane, hydroxychlorobactane glucoside, and hydroxychlorobactane glucoside laurate (Ng et al., 2010). The biosynthesis of carotenoids was also described in a GSB that has been isolated from Antarctica. Their precursors, isopentenyl diphosphate, and dimethylallyl diphosphate, are formed in the MEP pathway (also known as the methylerythritol phosphate pathway or the non-mevalonate pathway) from pyruvate and glyceraldehyde-3-phosphate. Other intermediates are geranyl diphosphate, farnesyl diphosphate, geranylgeranyl diphosphate, phytoene, ζ-carotene, lycopene and γ-carotene, precursor of the most important carotenoid chlorobactane (Figure 5) and its derivatives with different R groups: –H (1′,2′-dihydrochlorobactane), –OH (hydroxychlorobactane); glucose can also be attached to the hydroxyl group, with or without laurate. Chlorobactane is also a precursor of β-isorenieratene, from which isorenieratene can be formed (Figure 5). These two pigments are only present in brown members of the class *Chlorobia*, adapted to lower light intensity and higher wavelengths. It is not without interest that the conversion of γ-carotene to chlorobactane is catalyzed by the same enzyme (CruB) as the conversion of β-isorenieratene to isorenieratene—chemically it is a reaction of an identical functional group (Ng et al., 2010).

## 3 Photosynthesis and carbon assimilation

### 3.1 Light-dependent processes

The capture of the light quantum and excitation of the electron occur on bacteriochlorophyll *c*, *d*, or *e* molecules, which are deposited in the chlorosome. The excited electron relaxes back with simultaneous excitation of another chlorobial bacteriochlorophyll molecule. These molecules transfer the energy to the basal plate to bacteriochlorophyll *a*-795 molecules. It then moves on to the FMO complex, a designation derived from the names of the scientists Fenna, Matthews, and Olson. It is a complex of a pigment (bacteriochlorophyll *a*) and a protein with an absorption maximum at 808 nm. The energy is transferred from the FMO complex to the reaction center (Hauska et al., 2001).

**Figure 6:**
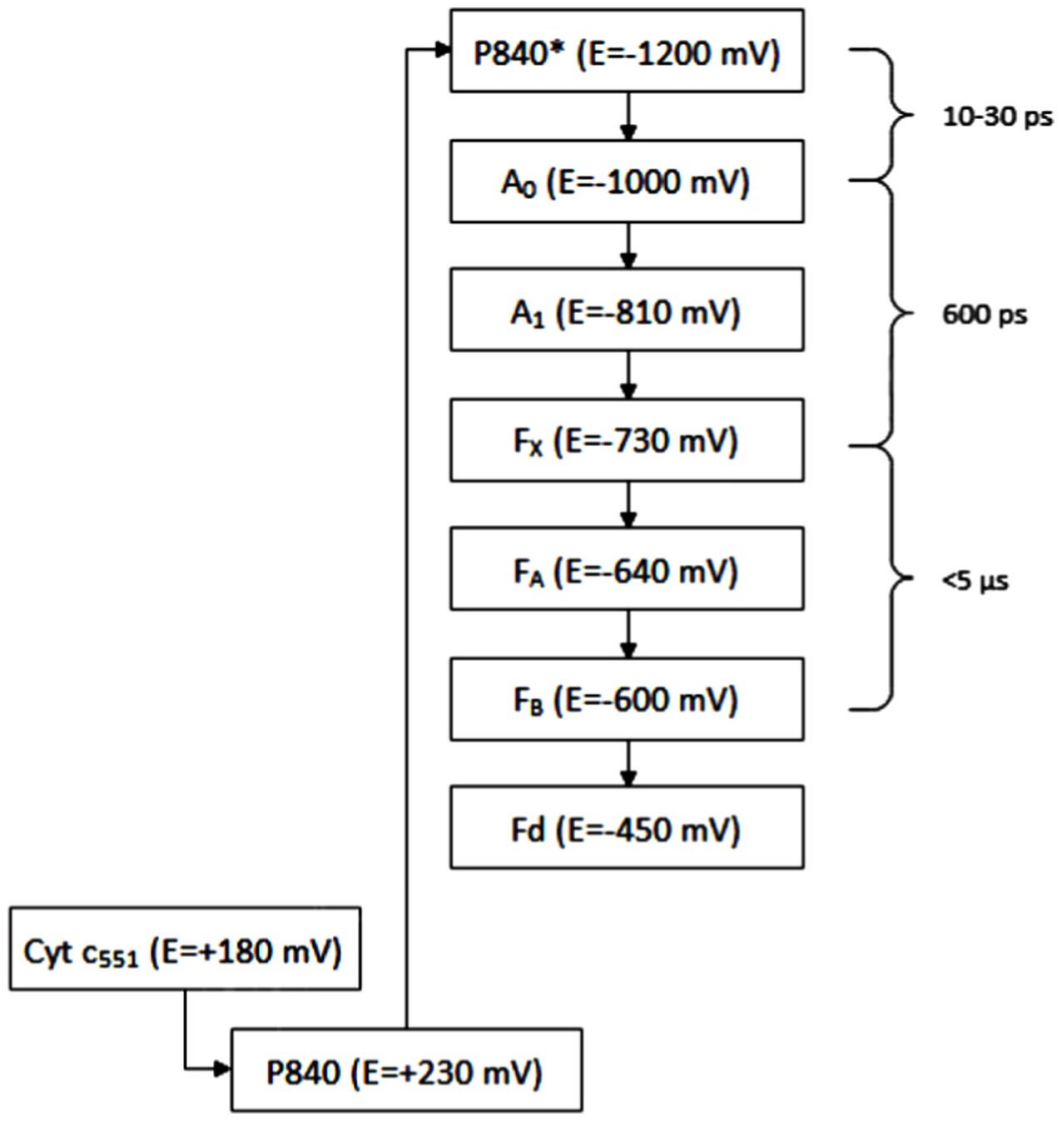
Electron transport chain: P840 is photosystem with maximum absorbance at 840 nm, A_0_, A_1_ means carriers of electrons, F_X_, F_A_, and F_B_ means iron–sulfur clusters, Fd means ferredoxins, and Cyt is cytochrome.

The FMO protein is a trimer, each of its subunits binds seven molecules of bacteriochlorophyll *a*. It is water soluble, which is unusual for protein complexes with bacteriochlorophylls. It is only found in GSB. The efficiency of energy transfer from chlorosomal carotenoids to the reaction center in *Cba. tepidum* was 23%; the major limiting step was the transfer from the FMO complex to the reaction center with the efficiency of 35%. Based on sequence similarity, it was suggested that the FMO protein is evolutionionary related to the reaction center protein PscA (Olson, 2004). It has already been shown how the FMO trimer associates with the photosynthetic reaction center (Chen et al., 2020). The reaction center is considered to be a homolog of photosystem I (PS I) in oxygenic phototrophs, but PS I is a heterodimer, whereas the reaction center of GSB is a homodimer with protein subunit composition [(FMO)_3_(PscA)_2_PscBCD]_2_ (Figure 6), containing eight molecules of bacteriochlorophyll *a* and two molecules of chlorophyll *a* for each molecule of PscA (Hauska et al., 2001). The reaction center of GSB has a homodimeric architecture with a symmetric distribution of pigments, suggesting two identical branches of electron transport chains (Gorka et al., 2021). The photosystem P840 is formed by a pair of bacteriochlorophyll *a* molecules. The excitation energy generally comes to it from bacteriochlorophyll *a*-837. Excited P840 is capable of charge separation: as the primary donor, it passes an electron to the carrier A_0_ (primary acceptor) and acquires a positive charge itself. Energy transfer to P840 and charge separation takes 25 ps; it is not clear whether the limiting step is the transfer of energy from bacteriochlorophyll *a*-837 to P840 or charge separation. The electron is then transferred to the secondary acceptor A_1_, then to the Fe–S center F_X_, and finally to PscB with the Fe–S centers F_A_ and F_B_. All of these three Fe-S centers can reduce ferredoxin, which can further reduce various metabolites, either directly or with an intermediate electron carrier such as NADH, NADPH, or FADH_2_. The sequence of the electron transport chain is schematically illustrated in Figure 6, including indicative values for the electrochemical potential of each compound and the times of individual reactions (or of groups of consecutive reactions in cases where partial reaction times could not be determined). The A_0_ carrier in GSB has been identified as chlorophyll *a*-670 (Hauska et al., 2001).

**Figure 7:**
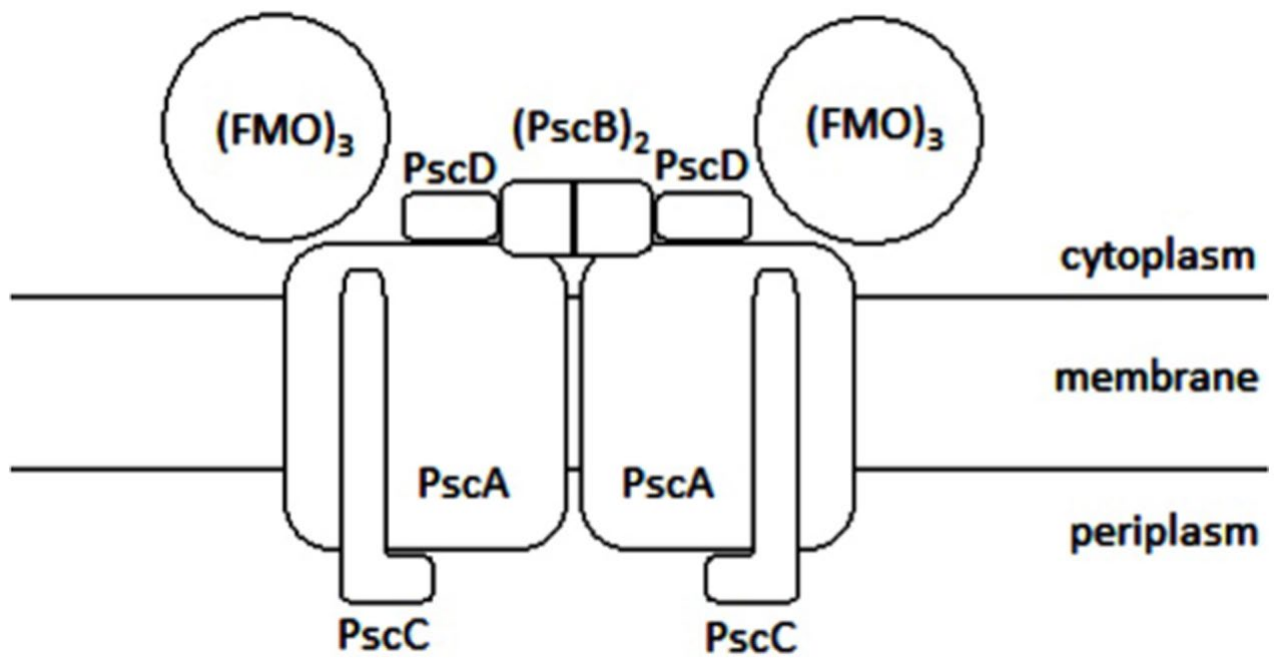
Schematic illustration of the photosyntetic reaction center of GSB: FMO is the abbreviation for Fenna, Matthews, and Olson complex, Psc depicts photosynthetic center proteins.

Baymann et al. (2001) reported that chlorophyll has a higher reducing power than bacteriochlorophyll and thus contributes to efficient electron transport toward the Fe-S centers with lower potential. The A_1_ carrier is phylloquinone. In other groups of phototrophic organisms, other molecules with the same function can be found. The localization of the individual members of the electron transport chain in the reaction center (Figure 7) is following: the PscC protein binds to cytochrome *c*-551, the PscA protein binds to P840, A_0_, A_1_ and F_X_, and the PscB protein binds to F_A_ and F_B_.

The description above refers to non-cyclic electron transport. Cyclic electron transport has only been predicted to date. The possible mechanism is the following: phyloquinone is sometimes reduced to phylloquinol, which may reduce menaquinone to menaquinol. Menaquinol can be reoxidized by Rieske Fe–S cluster, from which are the electrons transferred to cytochrome *c*-551 and back to P840 in the reaction center. This mechanism can only work if phylloquinol is mobile (Hauska et al., 2001).

**Figure 8:**
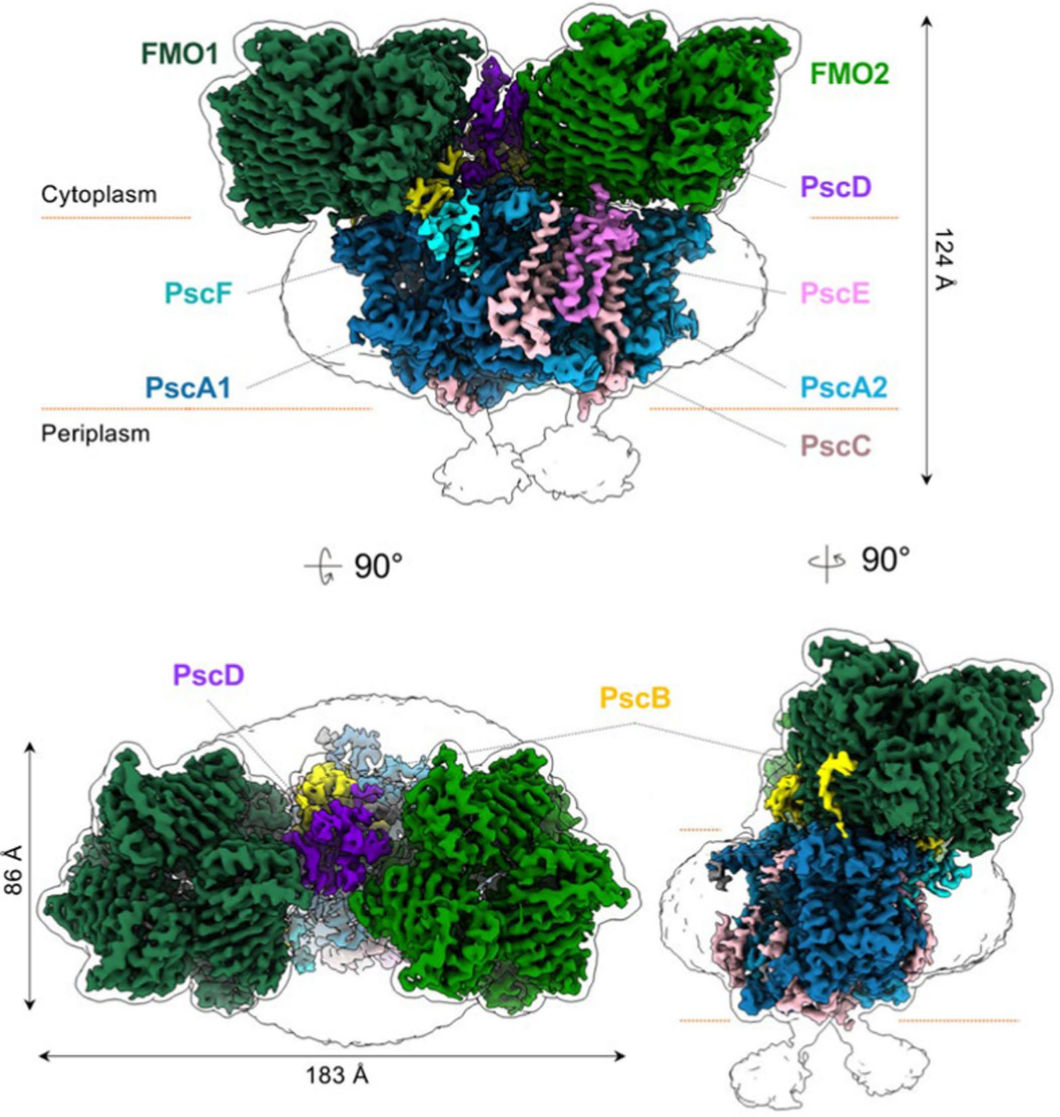
Reconstruction models of the reaction center-FMO_2_ photosynthetic supercomplex of *Cba. tepidium*. Three-dimensional cryogenic electron microscopy density map of the reaction center-FMO 2 assembly. The color codes are: FMO1—dark green; FMO2—forest green; PscA1—blue; PscA2—light blue; PscB—yellow; PscC—light pink; PscD—purple; PscE—magenta; and PscF—cyan. A 6 Å^−1^-filtered surface envelope (1.0σ) is overlaid over the density of the protein supercomplex (3.6σ). Horizontal dashed lines (orange) indicate membrane boundaries. The text was adapted from and the image was taken from Puskar et al. (2022).

The structure of the intact reaction center-FMO apparatus from *Cba. tepidum* became recently available from cryogenic electron microscopy at a resolution of 2.5 Å (Chen et al., 2020). Furthermore, the photosynthetic supercomplex consisting of the reaction center proteins and FMO was purified from *Cba. tepidum* and its high-resolution structure has been determined using single-particle cryogenic electron microscopy (Puskar et al., 2022). The purified supercomplexes revealed different stoichiometries of reaction center and FMO proteins. Moreover, the cryogenic electron microscopy reconstructions suggest that the reaction center can host at most two FMO trimer complexes on its cytoplasmic surface (Puskar et al., 2022). A cryogenic electron microscopy-based reconstruction of the photosynthetic supercomplex from *Cba. tepidum* is shown in Figure 8.

**Figure 9:**
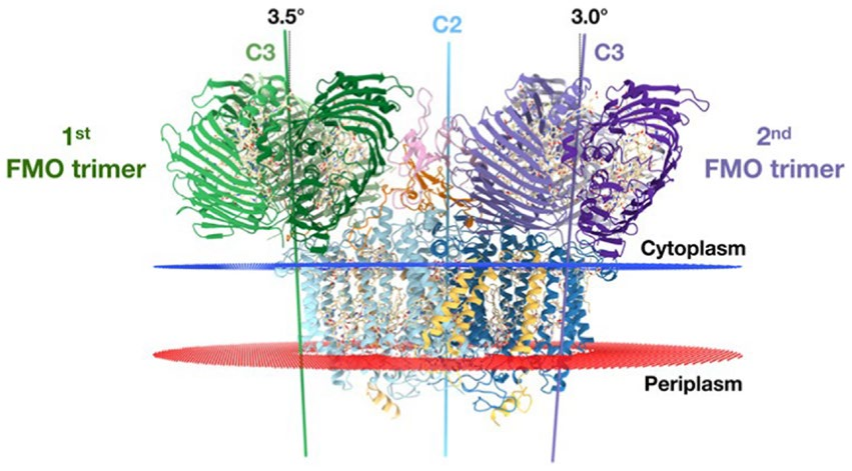
The reaction center [two PscA subunits are related by a 2-fold symmetry axis (blue line) (C2)] perpendicular to the membrane plane. The threefold symmetry axes (C3) of the first and second FMO trimer are indicated as green and purple lines, respectively. From this side view, the C3 axes of the first and second FMO trimer form a 3.5° and 3.0° angle on the cytoplasmic membrane normal. Text modified from and figure taken from Xie et al. (2023).

Recently the asymmetrical arrangement of the two FMO trimers has been revealed (Xie et al., 2023). A model of the asymmetrical arrangement of the two FMO complexes above the reaction center is shown in Figure 9.

Ferredoxin was successfully reoxidized by ferredoxin-NADP^+^ reductase from spinach. However, genes for such a reductase were not found in *Cba. tepidum*, so the mechanism of its reoxidation was further investigated. Seo and Sakurai successfully isolated ferredoxin-NAD(P)^+^ reductase from *Cba. tepidum* and studied its activity (Seo and Sakurai, 2002). It was able to reduce the artificial electron acceptor DPIP (2,6-dichlorophenol-indophenol) using both NADP^+^ and NAD^+^. At carrier concentrations below 0.5 mmol L^−1^, it was more active with NADP^+^, but at higher concentrations, it was more active with NAD^+^; thus, high concentrations of NADP^+^ appear to inhibit the reaction. Based on the sequence of the first 25 N-terminal amino acids, the corresponding ORF was found in the genome. The reductase was named TRLP (thioredoxin reductase-like protein) based on its sequence similarity to thioredoxin reductases from various bacteria (e.g., *Escherichia coli*, *Rickettsia prowazekii*, *Bacillus subtilis*, and *Bacillus halodurans*; Seo and Sakurai, 2002).

It has been proposed that GSB use a ferredoxin: NAD^+^ oxidoreductase of the RNF type, which works with the energy of the membrane gradient of Na^+^ concentration. Such enzymes are known from various heterotrophic bacteria, for example in connection with anaerobic fermentation (gradient generation) or with nitrogen assimilation and synthesis of Fe–S centers (gradient consumption). When the gradient is consumed, flavodoxin, which is a variant of ferredoxin with a reduced potential, tends to be reduced. In this study, the complete operon carrying *rnf* genes was found in some GSB, e.g., *Cbi. limicola*, *Cbi. luteolum*, *Cbi. phaeovibrioides*, and *Chp. thalassium*, *Prosthecochloris* spp., most of them being marine representatives. The operon showed 46–81% identity with the corresponding operon in the heterotrophic bacterium *Acetobacterium woodii*, known for having RNF enzymes. On the contrary, this operon was not found at all in the GSB *Cba. tepidum*, *Cba. limnaeum*, and others. Transcription of the mentioned operon was confirmed by RT-PCR. Thanks to a sufficient level of transcription, it was possible to observe the activity of RNF directly during ferredoxin (or, respectively, its low-potential form—flavodoxin) oxidation and NAD^+^ reduction. Previous experiments confirmed that this event is light-dependent. In addition to RNF, a cytoplasmic ferredoxin:NAD^+^ oxidoreductase, independent of the membrane gradient of Na^+^ concentration, was found in *Cbi. phaeovibrioides*. In *Cba. limnaeum*, only this cytoplasmic form was found. The gene coding is not part of the *rnf* operon. RNF ensures a high representation of reduced ferredoxin compared to its oxidized form, which leads to increased oxidative stress in aerobic environments. Other enzymes also use the energy of the membrane gradient of Na^+^. In *Cbi. phaeovibrioides*, this is the case of oxaloacetate decarboxylase, methylmalonyl-CoA decarboxylase and pyrophosphatase. The membrane gradient of Na^+^ can also be used for transport processes. If a Na^+^ gradient can be used to drive ATP synthesis in these organisms is not yet known. However, it is possible that the membrane gradient of Na^+^ helps to conserve energy and cover fluctuations in light intensity during the day (Bertsova et al., 2019).

Baymann et al. (2001) studied the evolutionary relationship between the reaction centers of different phototrophs. Cyanobacteria and photosynthetic eukaryotes, which are equipped with plastids derived from cyanobacteria have two different reaction centers: photosystem I and II (Baymann et al., 2001). The reaction centers of other phototrophic prokaryotes can be divided into type I (analogous to photosystem I) and type II (analogous to photosystem II). Besides cyanobacteria, type I reaction centers can be found in GSB and heliobacteria, and type II reaction centers in PSB and green filamentous bacteria (*Chloroflexi*). Type I reaction centers are generally homodimeric with two domains (PscA in GSB); the exception is the heterodimeric photosystem I with one domain PsaA and the other PsaB. Another common feature of these reaction centers is that they consist of an outer (antenna) and an inner domain. Electron transport occurs through chlorophyll *a* and phylloquinone to the Fe–S centers, the first of which F_X_ lies on the axis of symmetry, is present in a single copy and is followed by F_A_ and F_B_. Consequently, the electron is transferred to ferredoxin. Based on multiple sequence attachments for selected helices of the antennal and nuclear domains of the reaction centers, it appears that the reaction center of GSB is the most evolutionary distant of all others, including the type II reaction centers. This is consistent with the phylogenetic remoteness of the *Chlorobi* phylum from other bacteria (Gupta, 2004; Gupta and Lorenzini, 2007). It can be speculated that the two different reaction center variants arose by gene duplication in an ancestor close to the separation of a group of heliobacteria from the others and subsequent divergent evolution (Sattley and Swingley, 2013), with each group of phototrophic bacteria except cyanobacteria losing one of them. Another possibility is that the genes for type II reaction centers have been transferred laterally between groups, but the exact direction of transfer remains unresolved (Baymann et al., 2001).

### 3.2 Sulfur metabolism

The main electron donor for GSB is H_2_S, which is oxidized to elemental sulfur (Holkenbrink et al., 2011). The ability to oxidize sulfur to sulfate by some GSB was also reported. Besides that, *Cbi. limicola* f. thiosulfatophilum and *Cba. parvum* also oxidize thiosulfate and tetrathionate (Di Nezio et al., 2021; Han et al., 2022). These bacterial species also have an unique ability of photochemical sulfur disporportionation: they break down sulfur into H_2_S and sulfite in the presence of light and without access to carbon dioxide (CO_2_), then the resulting sulfite and part of the H_2_S synproportionate to form thiosulfate (Van Vliet et al., 2021). Thiosulfate was even observed in these organisms as an intermediate in the oxidation of H_2_S to sulfur. The use of sulfite as an electron donor has not been described in GSB. It is considered that polysulfides, e.g., S_3_^2−^, are also formed during the oxidation of H_2_S (Brune, 1989).

Various enzymatic options for sulfur oxidation are known among the GSB. However, none of GSB harbor all these sulfur metabolization pathways. The basic enzyme for H_2_S oxidation in GSB is sulfide:quinone oxidoreductase (SQR). Based on their structure, these enzymes can be divided into six classes (I–VI). The SQR in GSB usually belong to classes IV and VI, sometimes also to classes III or V. Using H_2_S, they reduce quinones, in the case of GSB it is menaquinone, which is reduced to menaquinol. Another enzyme capable of oxidizing sulfide is flavocytochrome *c*, which transfers electrons to cytochrome *c*. Flavocytochrome *c* has two subunits, FccA (type *c* cytochrome) and FccB (flavoprotein). They can also use DSR (dissimilatory sulfite reduction) systems, which use menaquinone as an electron acceptor. DSR systems are also known to occur in sulfate-reducing bacteria, where they play a crucial role in directing reduction reactions. Based on the comparison of the phylogeny of the studied GSB according to rRNA and DSR systems, it can be concluded that the genes for DSR were obtained by horizontal gene transfer—either from other sulfur-oxidizing bacteria or from sulfate-reducing bacteria. Finally, the Sox enzyme system can be used for sulfur oxidation. It was originally described in *Paracoccus pantotrophus*, but its homologs are present in all thiosulfate-oxidizing GSB. One of its components, the SoxCD complex, is absent in GSB, therefore the DSR system is also involved in the oxidation of thiosulfate by the Sox system. Electrons are transferred from the Sox system to cytochrome *c* (Gregersen et al., 2011).

Menaquinol, which is produced by the SQR and DSR systems, is reoxidized to menaquinone by Rieske’s Fe–S cluster, which transfers electrons to cytochrome *c*. From cytochrome *c*, electrons are returned to the reaction center at P840 (Hauska et al., 2001). In order to use extracellular sulfur globules as an electron donor, close contact of cells and globules is usually required. *Chlorobaculum tepidum* cells were observed to move toward sulfur globules, attaching to the globules, and detaching from the globules. Based on the comparison with the same culture in the dark and with a terminated culture, it was concluded that *Cba. tepidum* is motile. Genetic analysis determined that type IV pili are responsible for its motility. In a phase contrast microscope, it was further observed that the globules do not have to form attached to the cells, they most often formed at a distance of around 4 μm, the largest observed distance was 8 μm. The formation rate of the globules did not depend on the distance from the cell. The globule size was typically 0.5–1 μm. Based on observations using a scanning electron microscope, the globules were smooth and round, only slightly deformed when in contact with the cells. There were fewer globules in suspension than cells, so more cells are thought to contribute to the formation of one globule. Cells also divide during cultivation, while globules tend to grow. By having the condensation core located outside the cell, it is ensured that the cell is not surrounded by sulfur (Marnocha et al., 2016).

During the formation and degradation of globules, the presence of polysulfides was recorded, which are apparently an important intermediate in the metabolism of elemental sulfur (Loka Bharathi, 2008; Kushkevych et al., 2018a,b). Polysulfides are produced in the periplasm (in case of Fcc, SQR, and Sox systems) or in cytoplasm (when using DSR systems). The mechanism of their export through membranes remains still unknown (Gregersen et al., 2011; Marnocha et al., 2016). Polysulfides can form cycles, which is consistent with the detected presence of cyclic S_8_ molecules in biogenic sulfur. These cycles aggregate rapidly, the critical size of the condensation nucleus is only 30 nm. Contrary to original assumptions, it was observed that cell growth and consumption of sulfur globules are not dependent on close cell-globule contact. Some cells grew without being in contact with the globules and some globules degraded even without cell contact. One plausible explanation is as follows: cells that adhere to globules break them down into polysulfides. They import part of the polysulfides and use them as electron donors, but some of them diffuse. These polysulfides can serve as a substrate for cells that are not attached to the sulfur globules (Maki, 2013). However, at least part of the cells must be attached to the globules: if the cells and globules are separated by a membrane, the cells cannot grow on this sulfur. Extracellular storage of sulfur globules enables the sharing of sulfur between different cells as well as species of sulfur-oxidizing bacteria (Overmann and van Gemerden, 2000). Sulfur in this form is also more available to sulfur-reducing bacteria, which convert it back to H_2_S, the preferred electron donor for GSB (Jørgensen and Nelson, 2004; Marnocha et al., 2016).

### 3.3 Metabolic pathways used for carbon assimilation

**Figure 10:**
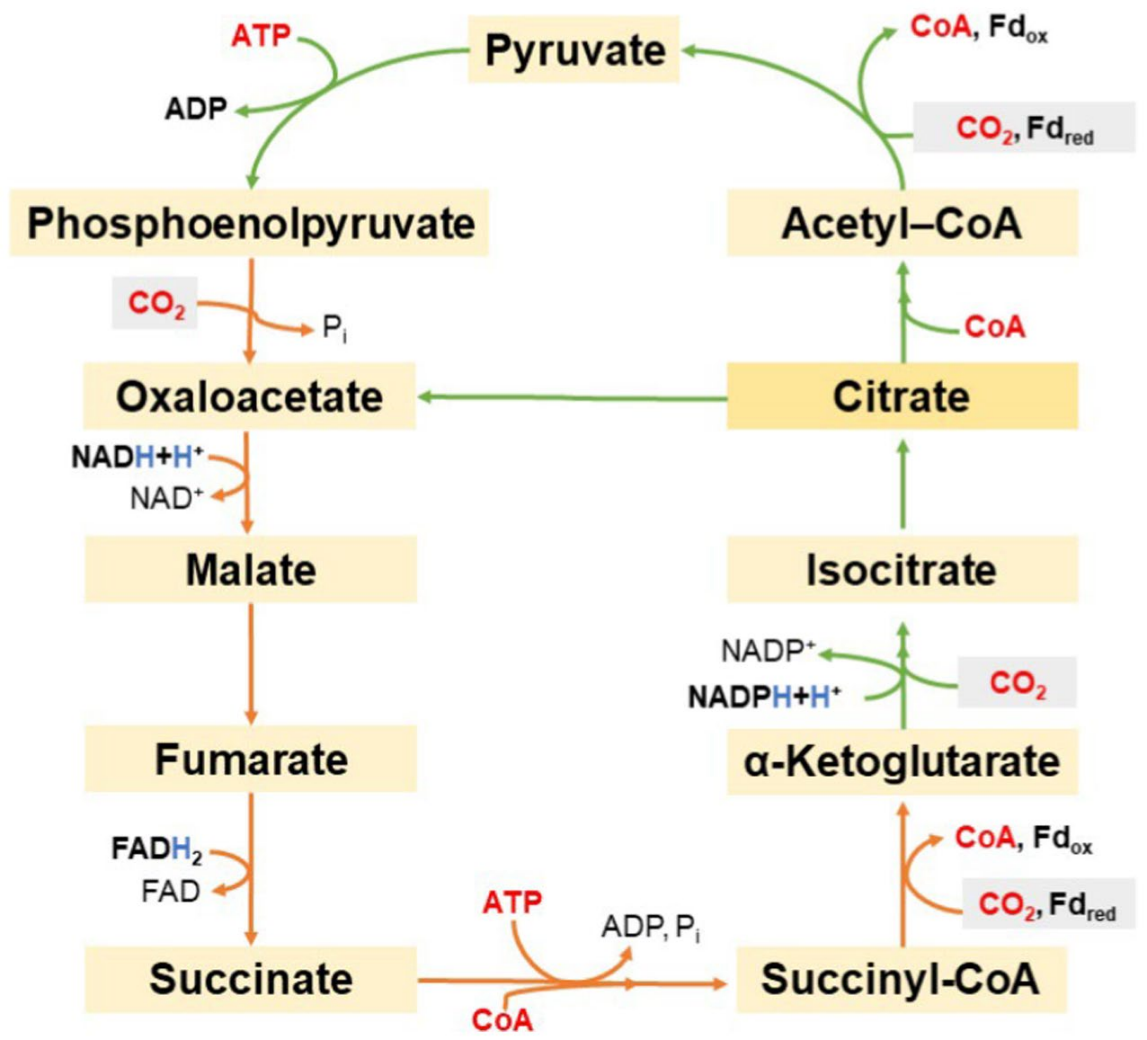
Schematic representation of the reductive tricarboxylic acid cycle.

The reductive pentose phosphate cycle, also known as the Calvin–Benson–Bassham (CBB), or as Calvin cycle, was described as the first CO_2_ fixation pathway that allows phototrophic organisms to assimilate CO_2_. The CBB cycle occurs in aerobic phototrophic, autotrophic organisms. Evans et al. described a completely different pathway for CO_2_ fixation that occurs was discovered in the GSB *Cbi. thiosulfatophilum*. Experimentally, Na_2_^14^CO_3_ was added to the *Cbi. thiosulfatophilum* culture. Then, the cell suspension was gradually removed over time, then it was dissolved in ethanol (80% final concentration) and analyzed by two-dimensional paper chromatography and radioautography. The content of labeled glutamate, glutamine, aspartate, succinate and phosphoric acid esters was evaluated. Similar tests (extended to include other intermediates of the reductive TCA cycle) were also performed with cell extract and with isolated enzymes that should participate in the cycle. The detected labeled metabolites allowed the conclusion that CO_2_ is processed in another aerobic CO_2_ fixation pathway. This cycle is now referred to as reductive citric acid cycle [sometimes also referred to as the reductive tricarboxylic acid (TCA) cycle or Arnon-Buchanon cycle]. Figure 10 illustrates the reductive TCA cycle (Evans et al., 1966). The cycle starts and ends with oxaloacetate. In the first part of the cycle, operating from oxaloacetate to citrate, 2 mol CO_2_ are fixed and 1 ATP is used. With the consumption of one additional ATP and coenzyme A, citrate is further converted to oxaloacetate and acetyl-CoA. The latter may be carboxylated twice more: first into pyruvate, then into oxaloacetate with the consumption of another ATP. However, there is also the possibility for a carboxylation of pyruvate to oxaloacetate using ATP (not shown in Figure 10).

Thus, during 1 cycle, four molecules of CO_2_ are fixed and two molecules of oxaloacetate are formed from one molecule of oxaloacetate, while one NADH, one FADH_2_, one NADPH, and two ATP are consumed. Intermediates of the mentioned cycle enter other anabolic pathways. For example, the proteogenic amino acid aspartate may be produced from oxaloacetate, glutamate from α-ketoglutarate, and alanine from pyruvate. The carboxylation of acetyl-CoA to pyruvate and the carboxylation of succinyl-CoA to α-ketoglutarate deserve special attention. Analogous reactions in the Krebs cycle are irreversible (Buchanan and Arnon, 1990). The reductive TCA acid cycle (Kumari, 2018) uses different enzymes and different co-factors: the fixation of CO_2_ requires the reduced form of ferredoxin (Li et al., 2021).

ATP citrate lyase (EC 4.1.3.8) catalyzes the conversion of citrate to oxaloacetate and acetyl-CoA in the reverse TCA cycle. This enzyme from *Cbi. limicola* underwent partial purification. It was observed that the utilization of substrates and the generation of products occurred in a balanced manner, and citrate breakdown followed the si-type mechanism. The enzyme activity was hindered by ADP and oxaloacetate, with the latter also impeding the growth of *Cbi. limicola* (Antranikian et al., 1982).

Later, a functional ribulose-1,5-diphosphate carboxylase was isolated from *Cbi. limicola*, upon which the reductive TCA cycle was contradicted. Catalysis of the reaction between ribulose-1,5-diphosphate and ^14^C labeled sodium bicarbonate was monitored in Tris–HCl and MgCl_2_. In contrast to the study by Evans et al. the assimilation was evaluated in a shorter time (first point already after 5 s) and it was found that the first labeled product is 3-phosphoglycerate. In addition, citrate ATP lyase, one of the enzymes of the reductive TCA cycle, could not be detected. These results would be more consistent with the use of the Calvin cycle (Sharma et al., 2020) rather than the reductive TCA cycle. The relative molecular weight of the studied enzyme was calculated as 3.61·10^5^ based on sedimentation comparison with other known proteins. The quaternary structure was evaluated by SDS-PAGE; thus, a single fraction with a relative molecular weight of 53,000 was obtained, leading to the conclusion that the enzyme has six subunits. Unlike other ribulose-1,5-diphosphate carboxylases, it does not contain any smaller subunit (Tabita et al., 1974).

However, later studies lean toward assimilation by the reductive TCA cycle in *Cbi. limicola*. The organism was cultured with ^14^C-labeled pyruvate and unlabeled CO_2_. In the cells, 20% of the carbon originated from pyruvate. No oxidation of pyruvate to CO_2_ was observed. Labeled compounds, including alanine, aspartate, glutamate, and glucose, were identified. Since pyruvate is not an intermediate in the Calvin cycle, this observation and the measured radioactivity support the theory that *Cbi. limicola* does not use the Calvin cycle (Fuchs et al., 1980b). 3-phosphoglycerate was not experimentally studied but it probably arises from pyruvate through gluconeogenesis. This conclusion was supported by another similar experiment, where labeled propionate was used instead of pyruvate. The latter was assimilated, probably via propionyl-CoA, methylmalonyl-CoA, and succinyl-CoA. Labeling of the individual carbon atoms in alanine, aspartate, and glutamate depend on the labeled position in the propionate also indicates the use of the reductive TCA cycle rather than the Calvin cycle (Fuchs et al., 1980a). Similar experiments were also performed using *Cbi. thiosulfatophilum* with non-radioactively labeled ^13^CO_2_, while the results were evaluated using NMR spectroscopy. This method provides signals for a selected nuclide with an odd number of nucleons, in this case ^13^C. The intensity of the signal depends on the ^13^C content and its position (so-called chemical shift) on the chemical environment of the respective atom, thanks to which one can distinguish which of the carbons in the molecule are labeled. *Cbi. thiosulfatophilum* was cultured on Larsen’s medium containing both Na_2_S and Na_2_S_2_O_3_ as sulfur sources and NaH^13^CO_3_ (enriched to 69% ^13^C) as carbon source; a second carbon source, unlabeled sodium acetate, was also added. Harvested cells were fractionated and selected metabolites were characterized by ^13^C NMR. Specifically, they were alanine, glycine, aspartate, glutamate, tyrosine, uridine, and guanosine. The enrichment of their individual carbons was calculated and compared to that predicted based on known metabolic pathways. These data were in agreement with the assumption that the assimilation takes place in the reductive TCA cycle, and the synthesis of other substances then in the assumed pathways, e.g., the Krebs cycle. On the contrary, it was obvious from the measured spectrum for ribose that this molecule could not have been formed in the Calvin cycle (Portis and Parry, 2007). This can be regarded as evidence indicating the exclusive utilization of the reductive TCA cycle for assimilation (Paalme et al., 1982). After sequencing the genome of *Cba. tepidum*, the gene for ribulose-1,5-diphosphate carboxylase was found, or only for the ortholog of its large subunit. However, CO_2_ fixation was not observed for the purified enzyme, and mutants in which this gene was knocked out had a standard phenotype (Eisen et al., 2002).

### 3.4 Assimilation of organic substances

In addition to photolithoautotrophic growth, mixotrophic growth has also been observed in GSB. The carbon source may be an organic molecule, for example acetate, but it is still necessary to supply the carbon source CO_2_, an electron donor (most often H_2_S) and light energy (Manske et al., 2005). Most of the fixed CO_2_ is directed to glucose-based polysaccharide and organic acids production. The addition of fluoroacetate to a culture of *Cbi. thiosulfatophilum* (strain 8346) generally inhibited CO_2_ fixation, but at the concentration lower than 1 mmol L^−1^ it increased the accumulation of α-ketoglutarate. CO_2_ fixation was completely inhibited by the addition of 0.1 mmol L^−1^ arsenite. Malonate, which inhibits the oxidative variant of the tricarboxylic acid cycle, caused a slight increase in CO_2_ uptake and a large increase in polysaccharide accumulation in *Cbi. thiosulfatophilum* (Cole and Hughes, 1965).

Furthermore, the assimilation of several other substances was tested in the presence of thiosulfate or H_2_ as an electron donor. Pyruvate was assimilated. In case of succinate, assimilation varied greatly in different bacterial suspensions. N_2_ did not affect metabolism in any way, but 0.05% ammonium chloride completely inhibited acid accumulation without affecting CO_2_ consumption. After the addition of fluoroacetate, which inhibits aconitase, there was no accumulation of citrate, which may indicate the absence of the oxidative tricarboxylic acid cycle. Accumulation of α-ketoglutarate instead of isocitrate can be explained by a shift in the balance in favor of α-ketoglutarate, possibly also through the inhibition of isocitrate dehydrogenase by fluoroacetate (Sirevåg and Ormerod, 1970).

Although GSB use the reductive TCA cycle to assimilate carbon, they can also operate the oxidative variant, Krebs cycle, which was proved by the following experiments. In *Cba. tepidum*, it was observed that the addition of pyruvate improves its growth by 20% and the addition of acetate by up to 50%. Neither acetate nor pyruvate excretion was observed during autotrophic growth, whereas 0.2 mmol L^−1^ acetate was excreted in mixotrophic growth on 20 mmol L^−1^ pyruvate. During mixotrophic growth on pyruvate or acetate labeled at various positions with ^13^C, the occurrence of labeled bacteriochlorophyll *c* was observed. Labeling was evaluated on the basis of MALDI–TOF. Furthermore, experiments were carried out with fluoroacetate, which successfully inhibited autotrophic growth and mixotrophic growth on pyruvate (in the latter case, however, weak growth was observed after increasing the concentration of pyruvate). In contrast, the addition of fluoroacetate had no significant effect during mixotrophic growth on acetate. Acetate is thought to be assimilated by acetyl-CoA synthetase. Since the assimilation of fluoroacetate practically did not occur, it is probably an unsuitable substrate for this enzyme. On the basis of the proportion of labeled carbon in bacteriochlorophyll *c*, a hypothesis was created that acetate is assimilated both by the reductive and oxidative tricarboxylic acid cycles, while pyruvate only by the reductive TCA cycle. This hypothesis is also confirmed by the experiment with fluoroacetate. If the overwhelming majority of pyruvate is assimilated by the reductive TCA cycle, then acetyl-CoA is formed by the breakdown of citrate with the enzyme aconitase. However, fluoroacetate inhibits aconitase, making it impossible for the cell to form acetyl-CoA. The activity of all enzymes specific for the reductive TCA cycle was observed, while the genes for enzymes specific for the oxidative cycle are either completely absent (pyruvate dehydrogenase, α-ketoglutarate dehydrogenase) or have a reduced level of expression (citrate synthase, succinate dehydrogenase). Thus, it can be concluded that in *Cba. tepidum* only a branch of the citric acid cycle oxidatively operates to form α-ketoglutarate. All other intermediates are only formed by the reductive TCA cycle, and therefore generated by consuming CO_2_. This is consistent with the already known fact that GSB do not grow heterotrophically (Tang and Blankenship, 2010).

### 3.5 Carbohydrate metabolism

The growth of GSB on glucose has not been observed. Accordingly, neither hexokinase nor glucose transporter genes were found in the genome of *Cba. tepidum*. Glucose is synthesized only inside the cells by the process of gluconeogenesis (Eisen et al., 2002; Tang et al., 2011). GSB accumulate polysaccharides during phototrophic growth, that are either of autotrophic or mixotrophic origin. However, more polysaccharide accumulates during mixotrophic growth. This polysaccharide was identified as a polymer of glucose in the genus *Chlorobium*. During subsequent cultivation in the dark, the cells consume the polysaccharide and release organic acids into the medium. When a culture of *Cbi. thiosulfatophilum* strain 8327 was incubated with NaHCO_3_, acetate, and thiosulfate for 3.5 h, an increase in polysaccharide concentration from 70 to 122 μg mL^−1^ was observed. Ultrathin sections were then made from these cells for electron microscopy and stained specifically for polysaccharide. In the electron microscope, dozens of colored granules were observed in each cell, which had an approximately uniform size (less than 30 μm) and consisted of smaller grains. The increase in their cell number after the incubation was in accordance with the results of the biochemical analysis of the polysaccharide consumption. The size of the individual granules did not change. In another experiment, cells were first incubated with ^14^CO_2_ in phototrophic mode for 4.5 h, then washed and incubated in the dark in the presence of unlabeled CO_2_ and thiosulfate for 12.5 h resulting in a degradation of polysaccharide. The vast majority of labeled carbon was found in the supernatant after centrifugation, of which 85% was in the form of lower fatty acids (80% of which was acetate, 17.5% capronate and 2.5% propionate). Unlike the bacterium *Rhodospirillum rubrum*, there was no formation of labeled formate. As for the remaining 15% of labeled carbon, the most abundant form was succinate. Last but not least, cells with accumulated labeled polysaccharide were cultured in the absence of both thiosulfate and any other common electron donor, either in the light or in the dark. Less polysaccharide (23%) was consumed during incubation in light conditions. However, the radioactivity of the supernatant was three times higher in the case of cells cultured in the dark. Polysaccharide may not only serve as an energy source for heterotrophic nutrition, but also as an emergency electron donor for photosynthesis (Sirevåg and Ormerod, 1970).

The storage polysaccharide of *Cbi. limicola* was later identified as glycogen, which is a homopolymer of glucose with α-(1 → 4) bonds and frequent branching using α-(1 → 6) bonds, soluble in water. As is typical for prokaryotes, 1-ADP-glucose is used to initiate glycogen synthesis (Levytska and Gudz, 2010). During cultivation in light in a medium containing H_2_S, NaHCO_3_ acetate, and pyruvate, the glycogen and glucose content in dry matter was measured. The glycogen content of the cells was almost always slightly higher than the glucose content, with approximately a 3-fold increase in both values between days 21 and 30. In addition, the cultivation of *Cbi. limicola* in wastewater from a distillery was studied. The wastewater was highly polluted with organic substances, but it did not contain glucose nor glycogen. Glycogen content in dry matter reached more than twice larger values than in the control. During heterotrophic growth in the dark, the glucose content in dry matter was similar in control and in wastewater, but the glycogen content decreased to 0.2% in wastewater. In any case, light was an effective method of bioremediation of wastewater, reducing its biological O_2_ consumption and H_2_S content (Saeed et al., 2022).

## 4 Cultivation conditions

### 4.1 Culture media

To cultivate GSB as phototrophic microorganisms, the main components of the culture medium are various minerals. Considering the mechanism of their photosynthesis, the most important medium components are H_2_S and CO_2_. Other important elements are primarily nitrogen, usually supplied as an ammonium salt, and phosphorus, supplied as hydrogen phosphate or dihydrogen phosphate. Van Niel used a basic mineral medium of the following composition for both PSB and GSB: NH_4_Cl, K_2_HPO_4_, MgCl_2_, NaHCO_3_, and Na_2_S·9H_2_O, all to a final mass fraction of 0.1% and dissolved in distilled water. With this medium, experiments were performed to enrich a mixed culture with either GSB or PSB. The temperature was approx. 25°C. At pH lower than 8, an increase in GSB was observed within 3 weeks of inoculation with an environmental sample containing both PSB and GSB. The cultivation success of GSB from the mixed culture could be improved by increasing the Na_2_S concentration (up to 0.25%) and decreasing the pH (up to 7.5). Higher Na_2_S concentrations or lower pH were not investigated in this study (van Niel, 1932). The GSB also grew faster than the PSB, so their capture is easier if the grown culture is collected early (Van Gemerden, 1986; Oren and Garrity, 2021).

Some GSB species, historically referred to as *Cbi. thiosulphatophilum*, are capable of utilizing thiosulfate as an electron donor. During their cultivation, 0.1–0.2% thiosulfate can be added to the medium described above. In this way, they can be enriched from the mixed culture with thiosulfate utilizing GSB at the expense of the others (van Niel, 1971).

Other authors use van Niel’s medium as a basis, but adjust the concentrations and sometimes add other components. The Handbook of Microbiological Media (Atlas, 2010) recommends a modified Pfennig medium prepared by mixing the following solutions for GSB: solution A: basic mineral solution, contains CaCl_2_, KH_2_PO_4_, NH_4_Cl, KCl, and MgSO_4_; solution B: distilled water only; solution C: vitamin B_12_; solution D: trace elements, contains iron, boron, cobalt, manganese, zinc, molybdenum, nickel and copper; solution E: NaHCO_3_ (1.5 g L^−1^ of the resulting medium); solution F: Na_2_S·9H_2_O (2 g L^−1^ of the resulting medium). Another study recommends the addition of ferric citrate and resazurin to the basic mineral solution (Malik, 1984).

The solutions are sterilized either by autoclaving or by filtration (this applies to solutions C and E). To reduce the redox potential, it is recommended to remove dissolved O_2_ from all solutions by bubbling with N_2_ (or CO_2_ in the case of solution E). The resulting pH should be 6.8 ± 0.2 at 25°C, adjusted with HCl and Na_2_CO_3_. There are several variants with different proportions of mineral salts. For marine representatives, the addition of NaCl to a final concentration of 10 g L^−1^ is recommended (Atlas, 2010). MgCl_2_ is also sometimes used as a magnesium source (Kobayashi et al., 1983).

### 4.2 Physical conditions

For the cultivation of GSB, a temperature of 25–30°C and lighting with an intensity of 700–2,000 Lx with a classic light bulb are recommended. Under these conditions, at pH of 6.6 and Na_2_S·9H_2_O concentration in the range of 0.1–0.2%, GSB grow significantly better than PSB, which can be used to enrich GSB cultures obtained from the environment. The absorption maxima of chlorophylls are most often in the range of 720–760 nm, the author assumes that illumination with these wavelengths could improve cultivation results. In natural conditions, the light spectrum is mainly influenced by the height of the water column above the bacteria, as water absorbs mainly higher wavelengths, and the presence of other phototrophs in it. At specific locations, absorption spectra were measured at varying water depths, extending to a point where no discernible amount of light penetrated. Pigments were extracted from the collected water samples to determine their concentration and mutual representation, which provided information on the ecological groups of phototrophs present. Furthermore, the rate of H_2_S consumption was studied depending on the light spectrum in pure cultures of two GSB—*Cbi. limicola*, a green-colored representative and *Cbi. phaeovibrioides*, a brown-colored representative. The absorption spectra of intact cells are similar for both species, with a maximum in the red and a second peak in the blue region. *Cbi. phaeovibrioides* is better adapted to blue and green light, confirmed by the results of measuring H_2_S consumption and the absorption spectrum. On the other hand, *Cbi. limicola* was favored in white and red light. Both species performed better in red light than in white light, as expected from the absorption spectra of bacteriochlorophylls. The results are consistent with the data on the occurrence of bacteria in the water column that the green-colored GSB thrive better at shallower depths than the brown GSB, and they better tolerate the presence of PSB in the higher layers, since their absorption spectra are largely complementary (Vila and Abella, 1994).

The only thermophilic representative of the GSB is *Cba. tepidum*, which grows fastest of all GSB in the presence of thiosulfate (Jagannathan and Golbeck, 2009). In addition to basic minerals, sodium thiosulfate, EDTA, vitamin B_12_ and a mixture of minerals were added to the medium. Cultivation took place at a temperature of 47°C and a light intensity of 40 μmol m^−2^ s^−1^. The ratio of thiosulfate to H_2_S concentrations was further optimized. At high concentrations of H_2_S, when the redox potential fell below −330 mV, growth was inhibited. At a redox potential of −300 ± 20 mV, the optimal thiosulfate concentration was 12 mmol L^−1^. Although thiosulfate is the preferred electron donor, cultivation was also successful only on H_2_S, but it was necessary to maintain its concentration below 0.1 mmol L^−1^, redox potential around −300 mV and pH around 6.8. During the oxidation of H_2_S to elemental sulfur, the pH increased, while further oxidation of sulfur, on the contrary, decreased. Last but not least, bubbling and mixing, important elements of cultivation in a larger volume (scale-up), were optimized. More intense mixing ensures equal access of individual cells to resources, especially to light, but too intensive mixing is accompanied by large shear forces that can damage the cells. The best growth was achieved at a frequency of 300 rpm, which corresponds to a Reynolds number of 55.125 and a peripheral speed of 165 cm s^−1^ for the given stirrer. The presence of inclusions that change the flow direction and increase turbulence improved the growth rate. Bubbling the culture with gases was not optimal for growth, better growth and without form formation was obtained by supplying N_2_ and CO_2_ through surface gassing (Mukhopadhyay et al., 1999).

### 4.3 Isolation from the environment and selection

When isolating GSB from the environment, the Winogradsky column can be used (De Leon et al., 2023). This simulates the natural environment in the anaerobic zone at the bottom of water bodies and streams. Mud taken from the environment is enriched with a source of sulfur (CaSO_4_) and organic carbon (for example, the roots of aquatic plants from the locality) and incubated in light in a suitable bottle. Elements circulate between heterotrophic and autotrophic bacteria, so the whole system can last for a long time without the supply of any chemicals, only requiring light. Gradually, zones inhabited by different microorganisms are created according to the lighting gradient and redox potential. GSB are most abundant in the lower (anaerobic) and illuminated part of the column, where they become visible as green spots. These green spots can be isolated and GSB can be further enriched. Molisch’s column is similar Winogradsky column, to which a piece of animal tissue is added. It decomposes in an anaerobic environment much faster than plant tissue, which will also accelerate the growth of microorganisms (van Niel, 1971).

A method for isolation and purification of microorganisms is dilution to extinction (Mauerhofer et al., 2019; Hanišáková et al., 2022). The method can be improved by the incorporation of micromanipulation techniques and removing a single cell from the culture. As GSB are sensitive to O_2_, they cannot grow on the surface of solidified agar when cultured exposed to air. The deficiency can be overcome by mixing the culture into a warm, still liquid medium at a temperature of around 45°C. Cultivation can be carried out in test tubes filled with a mixture of paraffin and paraffin oil to prevent access to O_2_. This mixture is preferable to paraffin alone, which shrinks during solidification and does not cover the entire surface of the medium well. Another problem is that GSB and PSB may thrive better in a consortium than in a pure culture, and their cells often stick close together, making pure culture isolation even more difficult (van Niel, 1932).

An alternative option for purifying cultures of anoxygenic phototrophs involves the use of antibiotics. Two strains of *Cbi. limicola* and the PSB *Allochromatium vinosum*, *Thiocapsa roseopersicina*, and *Thiocapsa* sp. sensitivity to different antibiotics was determined. The bacteria were first cultured in liquid medium, then mixed into agarized medium, spread on Petri dishes, and cultured in an anaerobic N_2_ atmosphere. Since H_2_S evaporates from the agar thioacetamide was added instead. Susceptibility to antibiotics was determined by the disk diffusion method. *Cbi. limicola* was particularly sensitive to amoxicillin, which caused complete lysis, but also to erythromycin, nalidixic acid, and nitrofurantoin. *Cbi. limicola* was also sensitive to gentamicin and netilmicin to some extent, but PSB were much more sensitive to these antibiotics, while they were not very sensitive to amoxicillin. Neither oxacillin nor trimethoprim and sulfamethoxazole significantly inhibited any of the cultures used. For selected antibiotics, critical concentrations of antibiotics, which should correspond to the limit of the inhibition zone, were also determined for *A. vinosum* and both strains of *Cbi. limicola*. Of these, both strains of *Cbi. limicola* had the lowest critical concentrations of mitomycin C (0.41 and 0.44 μg mL^−1^) and penicillin G (1.72 and 0.98 μg mL^−1^). Furthermore, penicillin G did not inhibit *A. vinosum*. If, on the other hand, the inhibition of *A. vinosum* and selection of *Cbi. limicola*, streptomycin would be the most suitable. The authors point out that the critical concentrations determined in this way may not fully correspond to the minimum inhibitory concentrations during cultivation in a liquid medium (Nogales et al., 1994).

The use of antibiotics for the selection of phototrophs from consortia would also have other disadvantages: in addition to the need for a detailed study of the sensitivity of different strains to different antibiotics, it would involve the consumption of antibiotics and the risk of developing resistance. However, the method could be more practical than purification of cultures on solid soils, especially if we aimed to isolate a particular ecological group rather than a pure culture (Wahlund and Madigan, 1995). Resistance to antibiotics can also be used as a selection marker to determine the success of the transfer of genetic information. During the selection of *Cba. tepidum* cells, in which conjugation was successful, solid medium with thioacetamide was used. Before selection, however, it was necessary to choose a suitable antibiotic to which *Cba. tepidum* is naturally sensitive. The antibiotic in the studied concentration was mixed directly into a culture medium solidified with agar. A small proportion of cells were resistant to kanamycin and streptomycin, especially when cultured at 37°C. This is not the temperature optimum, but it was chosen for conjugation as the optimum of *E. coli* donor cells. On the contrary, high sensitivity was observed to ampicillin, chloramphenicol and tetracycline (Wahlund and Madigan, 1995).

## 5 Perspective of biotechnological application

### 5.1 Desulfurization of gases and wastewater

H_2_S in wastewater might be problematic because it is relatively toxic to aquatic animals, causes corrosion, inhibits methanogenesis, smells strongly, and has a high O_2_ consumption requirement for oxidation. Kobayashi et al. were, to our knowledge, the first to investigate the possible use of phototrophic bacteria for wastewater desulfurization (Kobayashi et al., 1983). In addition to desulfurization, phototrophic bacteria can also degrade mercaptans and lipids (Imhoff, 2006). *Rhodospirillaceae* spp., representatives of the purple non-sulfur bacteria, are common in the environment and can consume H_2_S, but they can only tolerate it in low concentrations and are therefore not very suitable for wastewater desulfurization. In an experiment by Kobayashi et al., a column was used consisting of two concentric acrylic tubes. Into the inner tube, a 40 W tungsten light bulb was inserted. A consortium of anaerobic phototrophic bacteria isolated from the environment was inoculated into the column into the space between the outer and the inner tube and formed a biofilm. Brown and green colored zones with different bacteria developed in the column, the brown ones were in the more illuminated parts of the column. The most common morphological type were immobile rods with extracellular elemental sulfur. At the inlet, GSB dominated, further down the column, purple non-sulfur bacteria gradually became predominant (Kobayashi et al., 1983). Purple non-sulfur bacteria were identified as *Rhodopseudomonas acidophila*, GSB as *Cbi. limicola*. Desulfurization was quite successful: efficiency was 81–95%. Desulfurization did not occur when the lights were turned off, confirming that it was provided by phototrophic bacteria (Hurse et al., 2008). A commercial method employing chemotrophic sulfide-oxidizing bacteria within a fixed-film bioreactor under regulated O_2_ conditions was published. Fixed-film or suspended-growth photobioreactors using anoxygenic phototrophic bacteria may be viable alternatives for economical H_2_S removal for example from biogas. These systems can operate for extended periods without needing a biomass separation step and can handle high and fluctuating sulfide loads (Frigaard, 2016).

The tubular arrangement of the photobioreactor was more efficient than the column, which may have contributed to more uniform illumination and better flow, which ensured uniform mixing of the suspension and good contact of the cells with the H_2_S. In contrast to the column, elemental sulfur was not detected at the outlet from the tube; it was converted to sulfate at a retention time of 24 h. For further optimization, the authors of the study suggest focusing on the geometric arrangement of the photobioreactor. When choosing the lighting, the absorption spectrum of the pigments of the respective phototrophs was not taken into account (Kobayashi et al., 1983).

An important source of wastewater with an increased content of H_2_S are oil refineries. Oil is commonly desulfurized by e.g., the Holmes-Stretford process, or stripping followed by the Claus process (Kohl and Nielsen, 1997). Both are chemical methods. H_2_S is oxidized using O_2_ to elemental sulfur and as elemental sulfur is solid, it is easier to separate than H_2_S it also loses its corrosive properties and can be used as a raw material for the production of sulfuric acid (Saeid and Chojnacka, 2014). The disadvantages of the chemical methods are corrosion, the necessity of regular replacement of catalysts, consumption of additives, and work at high temperatures and pressures. Henshaw and Zhu grew GSB in a film in Tygon tubes, which are transparent and impermeable to O_2_. In their study, *Cbi. limicola* bacteria were grown on Pfennig’s medium under an infrared lamp (Henshaw and Zhu, 2001). The well-grown culture was allowed to circulate through the tube for 3 days to allow the cells to adhere to its wall. Wastewater was then allowed to flow through the tube for 72–288 h. At an influent concentration of H_2_S up to 286 mg L^−1^ h^−1^, its 100% removal was achieved, with 92–95% converted to elemental sulfur. The content of sulfates and bacteriochlorophyll was also monitored. On the contrary, the possibilities of the geometric arrangement of the reactor and methods of lighting were not studied (Henshaw and Zhu, 2001). Hurse and Keller proposed another type of photobioreactor for wastewater desulfurization using GSB (Hurse and Keller, 2004). Again, they used biofilm, but scale-up of a photobioreactor with biofilm in the tubes would be too difficult: simply magnifying the reactor would affect the ratio of irradiated surface (where the biofilm grows) to volume. A biofilm growing on optical fibers or transparent panels could overcome this problem. The variant with panels was also tested experimentally using artificial wastewater, with composition based on Pfennig’s medium (Pfennig and Trüper, 1981; Overmann et al., 1992b). The wavelength of the light was chosen as an interval between 720 and 780 nm. Compared to previous studies with tubes, desulfurization efficiency has decreased, but energy efficiency has increased (Hurse and Keller, 2004; Cui et al., 2021).

Other raw materials in which H_2_S occurs as an impurity are oil and natural gas. They are also usually desulfurized chemically. Hydrodesulfurization is a chemical method that uses H_2_ for reduction of all forms of sulfur to H_2_S, which is afterwards removed by absorption into a suitable solution (Shafiq et al., 2022). Another option is to absorb up to sulfur dioxide from the flue gas. A consortium of GSB was isolated from the hot spring and tested for natural gas desulfurization. Since gas eliminates the problem of separation, the planktonic form of bacteria could be used. The bioreactor had a volume of 4.5 L, was bubbled with natural gas for 15 min and then saturated with CO_2_. Desulfurization took place for a total of 3 weeks, after each week samples were taken to determine the production of sulfur and bacteriochlorophyll. Although desulfurization was successful, it was less effective than in other studies using chemolithotrophic microorganisms (Sharifian-Koupaiee et al., 2022).

In addition to natural gas, biogas can also be a gaseous fuel, which is produced from various wastes by their gasification with the help of microorganisms, the main components of which are methane and CO_2_ (Černý et al., 2018; Kushkevych et al., 2018b; Struk et al., 2019). Even here, a problematic amount of H_2_S can occur (up to 3%, depending on the raw material). Struk et al. studied the desulfurization of synthetic biogas containing 70% methane, 29.5% CO_2_, and 0.5% H_2_S. The PSB *A. vinosum* and the GSB *Cbi. limicola* were compared. The bacteria were cultured photoautotrophically without the addition of organic substances. In a stirred batch reactor, the efficiency of desulfurization of synthetic biogas and the effect of H_2_S concentration and light intensity on it were monitored. After 7 days of incubation of both cultures with biogas, complete desulfurization occurred. Then H_2_S was added to a concentration of 1%. This added H_2_S was removed up to 100% in 2 days in the reactor with *A. vinosum* and up to 90% in 5 days in the case of *Cbi. limicola* (Struk et al., 2023). For the abiotic control, approximately 33% H_2_S was removed by absorption into the water. About a third of the CO_2_ in the biogas was consumed for photosynthesis, so both the proportion of methane in the biogas and the quality of the biogas increased. Desulfurization was also successful at increasing the H_2_S concentration. Both cultures grew best at a H_2_S concentration of 1%, but the process was also effective at 2%. An increased concentration provided enough electron donor for photosynthesis, thereby limiting light damage to the photosynthetic apparatus in the later stages of cultivation. Compared to the consortium of chemolithotrophs and cyanobacteria, which tolerate a H_2_S concentration not higher than 16 mg L^−1^, anoxygenic phototrophs allow working with a concentration of up to 100–150 mg L^−1^. At a light intensity of 10 kLx, desulfurization was more effective than at 25 kLx, when *Cbi. limicola* grew more slowly and *A. vinosum* stopped growing completely. An experiment with continuous desulfurization using *Cbi. limicola* was carried out as part of this study. The lag phase lasted 6 days, while the optical density practically did not increase and the consumption of H_2_S was also minimal. After that, H_2_S consumption began to increase, from the 10^th^ day 100% desulfurization was observed. In the later stages of cultivation, there was a decrease in efficiency, which could be due to the accumulation of biomass, which reduced light transmission. For practical use, a semi-continuous arrangement with regular biomass sampling could be more suitable. All experiments were performed sterile and inoculated with pure cultures, which could also be limiting in practice (Struk et al., 2023).

### 5.2 Microbial electrochemical cells

A microbial electrochemical cell enables connection of the microbial metabolism with an electrical circuit and generation of electric current (Logan et al., 2006; Hassan et al., 2021). At its anode, organic substances from the solution are oxidized by respiring bacteria, and the released electrons are transferred to the circuit (Lovley, 2011, 2012). Defined microbial consortia may be suitable for this purpose, for example, a consortium of fermentative *Clostridium cellulolyticum* and respiring *Geobacter sulfurreducens* successfully generated electricity by breaking down cellulose (Desvaux, 2005; Poddar and Khurana, 2011). Other consortia also include phototrophic microorganisms. A microbial electrochemical cell can be inspired by biogeochemical sulfur cycling, for example using a consortium of sulfur-reducing bacteria and GSB (Badalamenti et al., 2014). Badalamenti et al. examined a consortium of the GSB *Cbi. limicola* in planktonic form and the GSB *Geobacter sulfurreducens* forming a biofilm on the anode. *Cbi. limicola* assimilated CO_2_ in the light and accumulated glycogen. Cultivation took place with alternating darkness (16 h) and light (8 h) (Badalamenti et al., 2014). In the dark, *Cbi. limicola* consumed glycogen, which was fermented to acetate. Acetate could thus be oxidized by the bacteria *G. sulfurreducens* and the obtained electrons travel to the anode and further into the electrical circuit. When the consortium was cultivated in the dark, a current of 118 ± 16 μA was generated, but in the light it dropped to 61 ± 11 μA within 10 min. Dependence of electric current on light was not observed in monocultures of *G. sulfurreducens* or *Cbi. limicola*, which supports the model above. The glycogen content was also determined at each transition between dark and light. During the first and second light periods, the glycogen content of the consortium increased to a similar level, yet the current decreased in the following dark phase. In the case of *Cbi. limicola* monoculture, glycogen regeneration was much less efficient in the light phases. A possible explanation is that *G. sulfurreducens* cells transferred electrons not only to the anode, but also to elemental sulfur produced during photosynthesis, reducing it back to H_2_S. This allowed *Cbi. limicola* to be photosynthetically active for longer periods and to accumulate glycogen, while diverting some of the electrons away from the anode, so that a decrease in electrical current was observed. Overall, this study is groundbreaking in that a microbial electrochemical cell was assembled not requiring the supply of an organic substrate. As *G. sulfurreducens* is sensitive to O_2_, it was expected that better results would be obtained in the consortium with an anoxygenic phototroph than in previous experiments with an oxygenic phototroph (*Chlamydomonas*), which was confirmed (Badalamenti et al., 2014). Possible applications of microbial electrochemical cells include bioremediation of wastewater, whether municipal or industrial. The advantage is that there is no need to work with an excess of activated sludge. Due to the high load of wastewater with heavy metals and other toxic inorganic substances, it is necessary to select such microorganisms that survive in this environment. The advantage of GSB is a high tolerance to H_2_S. Strains that have been isolated from a polluted environment might be particularly suitable. A consortium of the following bacteria for a microbial electrochemical cell was also used:

- *Desulfuromonas acetoxidans*, a sulfur-reducing bacterium capable of reducing Fe^3+^, a strain resistant to heavy metals (Pfennig and Biebl, 1976);
- *Cbi. limicola*, a GSB, consortium of GSB with *Desulfuromonas acetoxidans* also known from nature (Kyndt et al., 2020);
- *Desulfuromusa*, reduces sulfur, nitrate, nitrite, and various metal ions (Mn^4+^, Fe^3+^, Cu^2+^, and Cr^6+^), resistant to the toxic effects of chromium (Finster et al., 1997);
- *Geobacter* sp., similar properties to the previous strain (Jiang et al., 2022).

Heterotrophic representatives were cultivated as pure cultures on diluted landfill filtrate in a microbial electrochemical cell. All of them generated electric current; the highest specific power was achieved with *D. acetoxidans*. This bacterium was further cultured on diluted wastewater from yeast production. Many impurities were successfully removed, for example nitrates, nitrites, elemental sulfur, but also sulfates and sulfites, the use of which as electron acceptors was not yet known for this bacterium. On the contrary, the concentration of bicarbonate increased, probably due to the complete oxidation of organic substances. Further experiments were carried out with the participation of *Cbi. limicola*. Microbial electrochemical cells can also be used to remove hydrocarbons from water (Fatehbasharzad et al., 2022). In a bioreactor using a naturally occurring microbial community from contaminated groundwater, not only hydrocarbons were removed, but also sulfates, which were reduced by sulfate-reducing bacteria. However, the effectiveness of removing sulfates was reduced by bacteria of the genus *Chlorobium*, which reoxidized H_2_S formed into sulfate and transferred electrons to the anode. The activity of these bacteria is considered undesirable by the authors, as they regenerate sulfate. The reduction of sulfate to H_2_S is referred to by the authors as sulfate removal, but it is not stated that H_2_S is a toxic compound and that it is also soluble in water, so it is not a more suitable form of sulfur than sulfate. On the other hand, modification of this bioreactor to produce elemental sulfur is being considered in the future (Tucci et al., 2021).

## 6 Summary

Anoxygenic photosynthesis is a metabolic process utilized by some bacteria in which they generate energy from light without producing O_2_ as a byproduct. In anoxic environments where O_2_ is scarce or absent, various toxic compounds may accumulate, posing a threat to living organisms. Anoxygenic photosynthetic bacteria possess unique metabolic pathways that enable them to utilize alternative electron donors, such as H_2_S, H_2_, or ferrous iron, instead of water, to fuel their photosynthetic process. These bacteria play a crucial role in detoxification processes by converting harmful compounds into less toxic substances. By harnessing light energy, these bacteria can drive biochemical reactions that reduce the concentration of toxic compounds in their surroundings. Our review explores the ecological significance of anoxygenic photosynthetic bacteria in anaerobic environments. Additionally, the potential applications of these bacteria in bioremediation strategies are discussed with regard to mitigating environmental pollution that has been caused by toxic compounds. Overall, the role of anoxygenic photosynthesis is vital for maintaining ecosystem health and anoxygenic phototrophs may offer many still unexplored potential avenues in environmental management and biotechnology.

## Author contributions

IK: Visualization, Writing – review & editing, Writing – original draft. VP: Writing – review & editing, Writing – original draft, Visualization. MV: Writing – review & editing, Writing – original draft. DD: Writing – review & editing, Writing – original draft. MA: Writing – review & editing. SK-MRR: Writing – review & editing, Writing – original draft.
